# FGF19/SOCE/NFATc2 signaling circuit facilitates the self-renewal of liver cancer stem cells

**DOI:** 10.7150/thno.56369

**Published:** 2021-03-05

**Authors:** Jingchun Wang, Huakan Zhao, Lu Zheng, Yu Zhou, Lei Wu, Yanquan Xu, Xiao Zhang, Guifang Yan, Halei Sheng, Rong Xin, Lu Jiang, Juan Lei, Jiangang Zhang, Yu Chen, Jin Peng, Qian Chen, Shuai Yang, Kun Yu, Dingshan Li, Qichao Xie, Yongsheng Li

**Affiliations:** 1 Clinical Medicine Research Center, Xinqiao Hospital, Army Medical University, Chongqing 400037, China.; 2 Department of Medical Oncology, Chongqing University Cancer Hospital, Chongqing 400030, China.; 3 Department of Hepatobiliary Surgery, Xinqiao Hospital, Army Medical University, Chongqing 400037, China.; 4 Department of Oncology, The Third Affiliated Hospital, Chongqing Medical University, Chongqing 401120, China.

**Keywords:** FGF19, self-renewal, SOCE, NFATc2, LCSCs

## Abstract

**Background & Aims:** Liver cancer stem cells (LCSCs) mediate therapeutic resistance and correlate with poor outcomes in patients with hepatocellular carcinoma (HCC). Fibroblast growth factor (FGF)-19 is a crucial oncogenic driver gene in HCC and correlates with poor prognosis. However, whether FGF19 signaling regulates the self-renewal of LCSCs is unknown.

**Methods:** LCSCs were enriched by serum-free suspension. Self-renewal of LCSCs were characterized by sphere formation assay, clonogenicity assay, sorafenib resistance assay and tumorigenic potential assays. Ca^2+^ image was employed to determine the intracellular concentration of Ca^2+^. Gain- and loss-of function studies were applied to explore the role of FGF19 signaling in the self-renewal of LCSCs.

**Results:** FGF19 was up-regulated in LCSCs, and positively correlated with certain self-renewal related genes in HCC. Silencing FGF19 suppressed self-renewal of LCSCs, whereas overexpressing FGF19 facilitated CSCs-like properties* via* activation of FGF receptor (FGFR)-4 in none-LCSCs. Mechanistically, FGF19/FGFR4 signaling stimulated store-operated Ca^2+^ entry (SOCE) through both the PLCγ and ERK1/2 pathways. Subsequently, SOCE-calcineurin signaling promoted the activation and translocation of nuclear factors of activated T cells (NFAT)-c2, which transcriptionally activated the expression of stemness-related genes (*e.g., NANOG*, *OCT4* and *SOX2*), as well as *FGF19*. Furthermore, blockade of FGF19/FGFR4-NFATc2 signaling observably suppressed the self-renewal of LCSCs.

**Conclusions:** FGF19/FGFR4 axis promotes the self-renewal of LCSCs *via* activating SOCE/NFATc2 pathway; in turn, NFATc2 transcriptionally activates FGF19 expression. Targeting this signaling circuit represents a potential strategy for improving the therapeutic efficacy of HCC.

## Introduction

Hepatocellular carcinoma (HCC), the third lethal cancer worldwide, is characterized by a high rate of recurrence and therapy-resistance 1, 2. Compelling evidence has demonstrated that small heterogeneous populations of liver cancer stem cells (LCSCs) with self-renewal properties are critical for the progression and therapy-resistance of HCC 2, 3. Self-renewal is one of the most important properties employed by the CSCs to sustain the proliferating capacity 4, 5. Understanding the mechanisms underlying the self-renewal of LCSCs will provide potential strategy to overcome the recurrence and therapy-resistance of HCC.

Fibroblast growth factors (FGF) signaling can promote self-renewing proliferation and inhibit cellular senescence in many tissues and organs 6, 7. As a member of the hormone-like FGF family, FGF19 shows high binding affinity with FGF receptor (FGFR)-4, which is predominantly expressed in liver 8, 9. Growing evidence indicates aberrant FGF19-FGFR4 signaling axis is a key driver in the development of HCC 8, 10, 11. Our earlier study and others indicated that FGF19 regulates a variety of functions of hepatocytes, such as bile acid synthesis, proliferation, epithelial-mesenchymal transition (EMT) and apoptosis-resistance in a FGFR4-dependent manner 12-15. In addition, previous study demonstrated that expressions of FGF19 positively correlated with undifferentiated state of human embryonic stem cells (ESCs) 16. However, whether FGF19 signaling is related to self-renewal characteristics of LCSCs is unknown.

Calcium (Ca^2+^)-mediated signaling pathways are involved in various cellular biological processes including proliferation, apoptosis, metastasis and self-renewal 17. Store-operated Ca^2+^ entry (SOCE), triggered by depletion of endoplasmic reticulum (ER) Ca^2+^, is the major route of Ca^2+^ influx for non-excitable cells including HCC cells 17, 18. Stromal interaction molecule (STIM)-1, as an ER Ca^2+^ sensor, is a key mediator of SOCE activation 19, 20. We previously reported that STIM1-mediated SOCE orchestrates HCC tumorigenesis under hypoxic conditions 21, whereas how SOCE is mobilized in LCSCs and regulates the HCC self-renewal remain vague. Herein, we sought to assess the effect of FGF19/FGFR4 signaling on STIM1-mediated SOCE and the self-renewal of LCSCs *in vitro* and *in vivo*.

## Results

### FGF19 is up-regulated in LCSCs and correlates with self-renewal related genes in HCC

We firstly enriched LCSCs of Huh-7, RFP/PLC/5 and MHCC97H cells *via* serum-free suspension 22. After cultured for 2 weeks, Huh-7 and RFP/PLC/5 cells formed compact round multicellular aggregates, while MHCC97H cells formed branching spheroids (Figure S1A). The high self-renewal capacity of spheroids cells was confirmed by sphere formation assay, clonogenicity assay, sorafenib resistance assay and tumorigenic potential assay (Figure S1B-E). Furthermore, comparing with the parental cells, spheroids exhibited increased mRNA levels of several stem cell markers, *e.g.*, pluripotency transcription factor Nanog, octamer-binding protein 4 (Oct-4), SRY-box transcription factor 2 (Sox2), CD133, c-Myc and aldehyde dehydrogenase1 (ALDH1), but showed decreased mature hepatocyte markers, such as albumin (ALB) and glucose-6-phosphatase (G6P) (Figure S1F). These results demonstrate that the *in vitro* enriched LCSC populations of Huh-7, RFP/PLC/5 and MHCC97H cells were successfully established.

**Figure 1:**
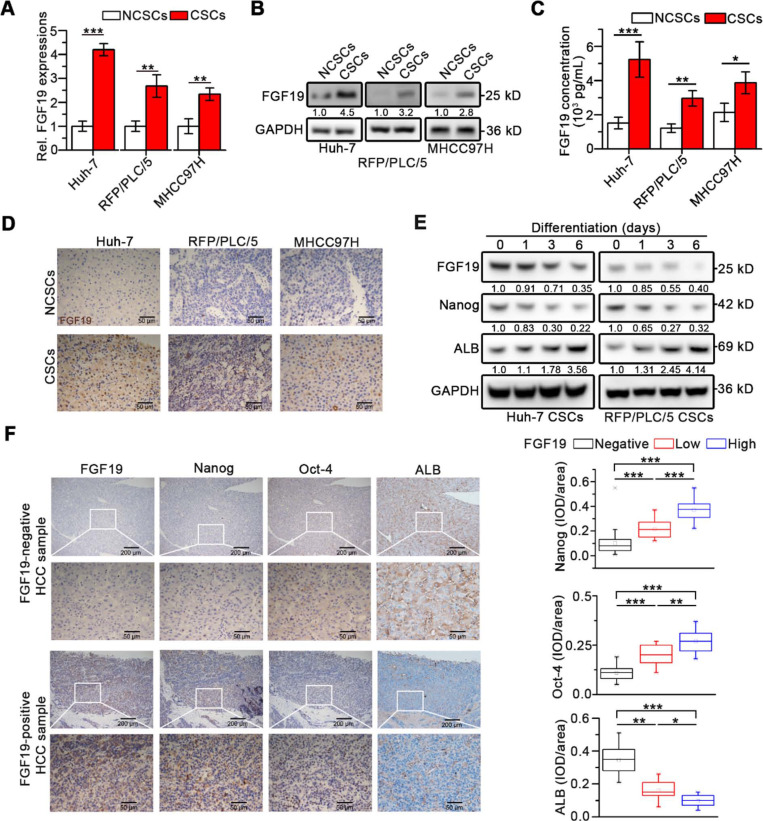
** Upregulated FGF19 is associated with enhanced self-renewal in HCC. (A**-**C**) FGF19 expressions in CSCs of Huh-7, RFP/PLC/5 and MHCC97H and corresponding NCSCs: (**A**) mRNA levels were measured by RT-qPCR, (**B**) protein levels were determined by WB, and (**C**) protein concentrations in cell supernatant were measured by ELISA. (**D**) Representative micrographs of FGF19 IHC analysis (400×) in xenograft tumors derived from LCSCs and NCSCs of Huh-7, RFP/PLC/5 and MHCC97H. (**E**) Dynamic expressions of FGF19, Nanog, and ALB were detected in LCSCs at different time points during differentiation. (**F**) Representative micrographs of FGF19, Nanog, Oct-4, and ALB IHC analysis (400×) in 20 HCC samples (left panel); and statistical analysis of integrated optical density (IOD) of FGF19, Nanog, Oct-4, and ALB against immunoglobulin G (right panel). Data are expressed as means ± SEM (n = 3). *p < 0.05, **p < 0.01, ***p < 0.001.

Interestingly, compared with corresponding non-CSCs (NCSCs), both FGF19 expression and secretion remarkably up-regulated in LCSCs (Figure 1A-C). Immunohistochemistry (IHC) analysis revealed high levels of FGF19 in xenograft tumors derived from CSCs compared with tumors from corresponding NCSCs (Figure 1D). During the differentiation of LCSCs, we observed a gradual decrease of FGF19 expression accompanied with the decrease of endogenous Nanog and the increase of ALB (Figure 1E). Next, IHC analysis of human HCC samples showed that FGF19 expression was positively correlated with expressions of Nanog and Oct-4, but negatively correlated with expressions of ALB (Figure 1F). These data indicate that FGF19 is highly expressed in LCSCs, and correlates with stemness-related genes in HCC.

### Knockdown of FGF19 attenuates self-renewal features of LCSCs

**Figure 2:**
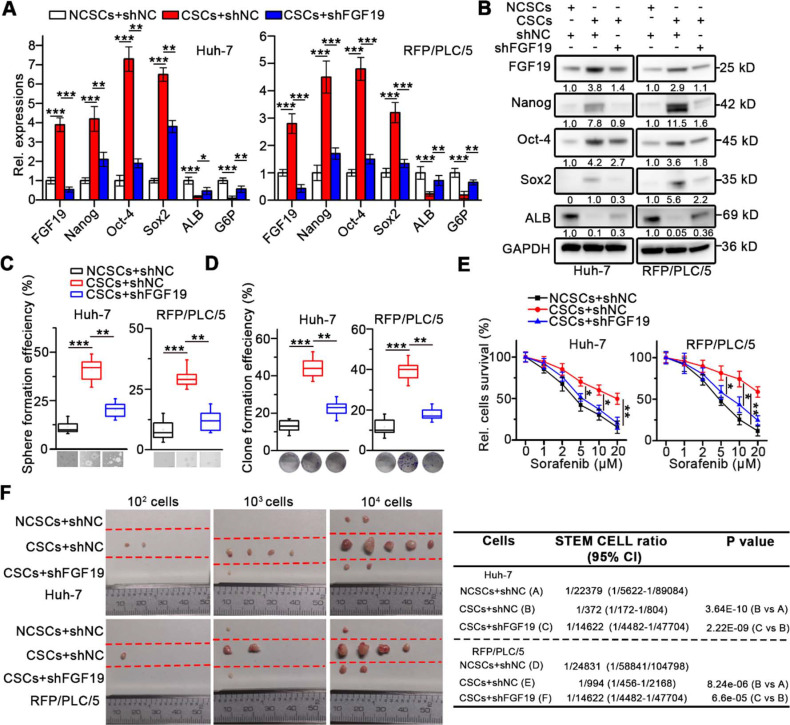
** Knockdown of FGF19 attenuates self-renewal in LCSCs.** After introducing negative control shRNA (shNC) or shRNA targeting FGF19 (shFGF19) into Huh-7 and RFP/PLC/5 cells, (**A**) RT-qPCR and (**B**) WB were applied to measure levels of FGF19, Nanog, Oct-4, Sox2, ALB and G6P in NCSC transfected with shNC, and in CSCs transfected with shRNA or shFGF19. (**C-F**) The effects of FGF19 silencing on self-renewal features of LCSCs were assessed by (**C**) sphere formation assay, (**D**) clonogenicity assay, (**E**) sorafenib resistance assay, and (**F**) tumorigenic potential assays* in vivo*. Data are expressed as means ± SEM (n = 3). *p < 0.05, **p < 0.01, ***p < 0.001.

To investigate the role of FGF19 on self-renewal of LCSCs, we knocked-down FGF19 *via* lentiviral introducing shRNA in the CSCs of Huh-7 and RFP/PLC/5 (Figure 2A-B). We found that stemness-associated genes including *NANOG*, *OCT4* and *SOX2* were significantly down-regulated, whereas mature hepatocyte markers, such as *ALB* and *G6P* were markedly up-regulated, when compared with control (Figure 2A-B). Consistently, FGF19 silencing significantly reduced the sphere and clone formation ability, as well as the sorafenib resistance of LCSCs (Figure 2C-E). Moreover, the growth of xenograft tumors generated from FGF19-deficient LCSCs was attenuated compared with control LCSCs (Figure 2F). These results indicate that FGF19 is required for self-renewal in LCSCs.

### FGF19 facilitates cancer stem cell-like properties in liver NCSCs

**Figure 3:**
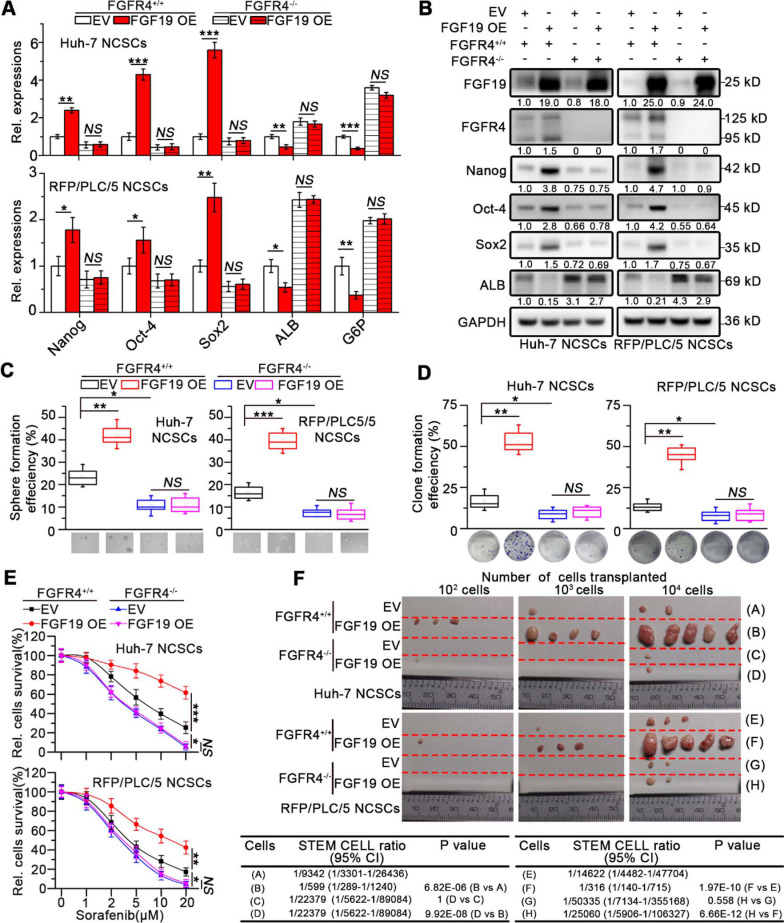
** FGF19 promotes self-renewal *via* FGFR4 in none-LCSCs.** (**A**) RT-PCR and (**B**) WB were applied to measure levels of Nanog, Oct-4, Sox2, ALB and G6P in FGFR4^+/+^ NCSCs transfected with empty vector (EV) and FGF19-overexpressing vector (FGF19 OE), as well as in FGFR4^-/-^ NCSCs. (**C-F**) The effects of exogenous FGF19 expression on self-renewal features of FGFR4^-/-^- and FGFR4^+/+^ in NCSCs of Huh-7 and RFP/PLC/5 were measured by (**C**) sphere formation assay, (**D**) clonogenicity assay, (**E**) sorafenib resistance assay and, and (**F**) tumorigenic potential assays *in vivo*. Data are expressed as means ± SEM (n = 3). *p < 0.05, **p < 0.01, ***p < 0.001, *NS* represents no significant difference.

To further validate the role of FGF19 in maintaining the stemness of HCC cells, we overexpressed FGF19 in the NCSCs of Huh-7 and RFP/PLC/5 by lentiviral vector. Ectopic expression of FGF19 upregulated stemness-associated genes (*e.g.*, *NANOG*, *OCT4* and *SOX2*), while reduced *ALB* and *G6P* levels (Figure 3A-B). Over-expression of FGF19 significantly promoted clone and sphere formation, as well as the resistance to sorafenib (Figure 3C-E). Furthermore, compared with the control group, FGF19 overexpression significantly enhanced the tumorigenic ability of NCSCs (Figure 3F). Our previous studies have shown that FGF19 promotes EMT and facilitates a survival response to ER stress *via* FGFR4 in HCC cells 12, 13. To determine the possible involvement of FGFR4 in FGF19-mediated self-renewal of LCSCs, we generated genomic FGFR4 knockout Huh-7 and RFP/PLC/5 cells by CRISPR-Cas9 system (Figure S2A-B). FGFR4 deficiency dramatically suppressed self-renew properties trigged by FGF19 in NCSCs (Figure 3A-F). Moreover, BLU9931, a specific FGFR4 inhibitor, also attenuated the FGF19-induced self-renewal properties in NCSCs of Huh-7 and RFP/PLC/5 (Figure S3A-E). These results demonstrate that FGF19 facilitates self-renewal properties* via* FGFR4 activation in HCC cells.

### Enhanced SOCE is involved in FGF19-promoted self-renewal of HCC cells

Recent evidence indicated that Ca^2+^ oscillation frequency positively correlated with the self-renewal potential in Hep-12 cells 23, we next wondered whether SOCE-mediated Ca^2+^ signal was involved in FGF19-mediated self-renewal activity. Firstly, high levels of phosphorylated Ca^2+^/calmodulin dependent protein kinase II (CaMKII) (Th286), usually activated by elevated intracellular Ca^2+^, were observed in LCSCs (Figure S4A). Moreover, blockade of SOCE by SKF-96365 significantly inhibited the sphere and clone formation capabilities of LCSCs *in vitro,* and suppressed the expressions of stemness-related genes (Figure S4B-D).

**Figure 4:**
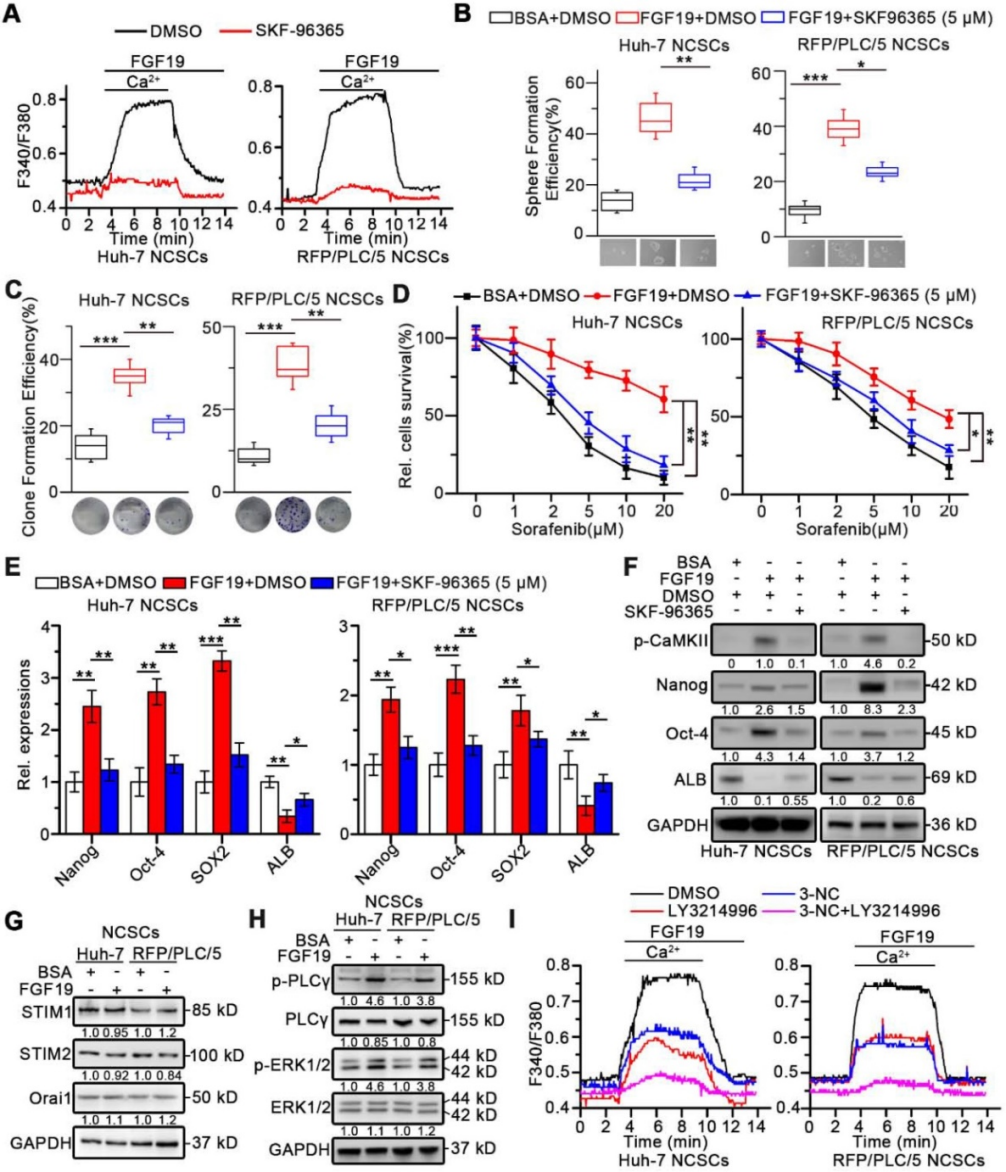
** Enhanced SOCE is critical for FGF19-promoted self-renewal of HCC cells.** (**A**) Huh-7 and RFP/PLC/5 NCSCs were pre-treated with DMSO or SKF96365 (5 µM), then Ca^2+^ mobilization in Fura-2-loaded cells respectively upon FGF19 (100 ng/ml) stimulation were measured and expressed as means ± SEM of 10 independent cells each group. (**B-D**) The effects of SKF-96365 (5 µM) on self-renewal features of FGF19 (100 ng/ml) treated-NCSCs were measured by (**B**) sphere formation assay, (**C**) clonogenicity assay, (**D**) sorafenib resistance assay. (**E**) RT-PCR and (**F**) WB were applied to measure levels of Nanog, Oct-4, Sox2 and ALB in FGF19 (100 ng/ml) treated NCSCs, in the presence of SKF-96365 (5 µM) or not. (**G**) SOCE related protein levels (STIM1, STIM2, and Orai1); and (**H**) protein levels of p-PLCγ, PLCγ, p-ERK1/2, and ERK1/2 were examined by WB in FGF19 (100 ng/ml) treated- or BSA (100 ng/ml) treated Huh-7 and RFP/PLC/5 NCSCs for 4h. (**I**) Cells were pre-treated with DMSO, 3-NC (20 µM), LY3214996 (2 µM), or 3-NC (20 µM) + LY3214996 (2 µM) for 2h, respectively; then Ca^2+^ mobilization in Fura-2-loaded cells respectively upon FGF19 stimulation were measured and expressed as means ± SEM of 12 independent cells each group. Data are expressed as means ± SEM (n = 3). *p < 0.05, **p < 0.01, ***p < 0.001.

Furthermore, both Huh-7 and RFP/PLC/5 NCSCs displayed a striking increase in Ca^2+^ influx in response to FGF19, which could be suppressed by SKF96365 (Figure 4A), demonstrating that SOCE mediates Ca^2+^ mobilization induced by FGF19 in HCC cells. More importantly, SKF-96365 remarkably attenuated the self-renewal activity in FGF19-OE NCSCs, including the sphere and clone formation (Figure 4B-C), sorafenib resistance (Figure 4D), and expressions of stemness-associated markers (Figure 4E-F). These observations indicate that enhanced SOCE is essential for FGF19-promoted self-renewal of NCSCs.

### FGF19 enhances SOCE through the PLCγ and ERK pathways in HCC cells

Interestingly, after FGF19 treatment, there were no significant changes on the expressions of STIM1, STIM2 and Orai1, which are key SOCE-related molecules (Figure 4G and S5A). Since FGF19-FGFR4 signaling can activate PLCγ-IP_3_R and ERK1/2 pathways 24, 25, both of which are critical for SOCE activation, such coordinating events lead us to speculate whether FGF19 enhances SOCE function *via* PLCγ-IP3R and ERK1/2 pathways in HCC. Indeed, FGF19 increased p-PLCγ and p-ERK1/2 protein levels (Figure 4H). The PLCγ inhibitor 3-NC or ERK1/2 inhibitor LY3214996 attenuated FGF19-dependent SOCE activation in Huh-7 and RFP/PLC/5 NCSCs. Noteworthily, combination of these two inhibitors almost completely blocked FGF19-triggered SOCE (Figure 4I and S5B). Given STIM1 oligomerization after ER Ca^2+^ store depletion is essential for SOCE 26, we introduced STIM1-mcherry chimeric vector in Huh-7 and RFP/PLC/5 NCSCs (Figure S5C). FGF19 significantly increased STIM1 puncta, which was restrained by BLU9931. In addition, combination of 3-NC and LY3214996 inhibited the aggregation of STIM1 induced by FGF19 stimulation (Figure S5C). These results indicate that FGF19 promotes SOCE *via* synergy of PLCγ and ERK1/2 pathways.

### NFATc2 nuclear accumulation is required for FGF19-dependent self-renewal

**Figure 5:**
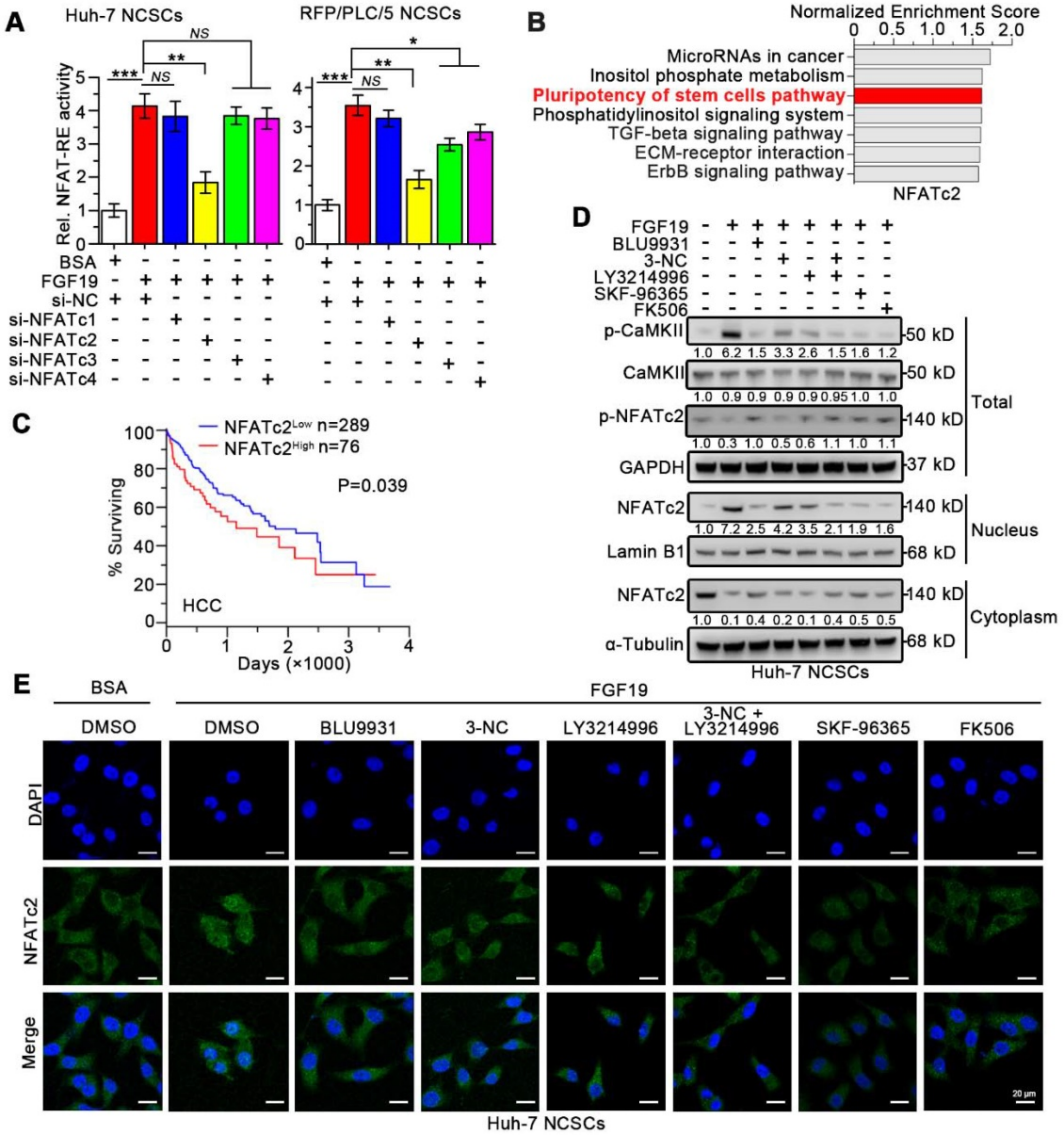
** FGF19 signaling facilitates dephosphorylation and nuclear translocation of NFATc2 in non-LCSCs.** (**A**) NFAT-RE luciferase activity was measured in FGF19 (100 ng/ml) treated Huh-7 and RFP/PLC/5 NCSCs transfected with si-NC, si-NFATc1, 2, 3, 4, respectively. (**B**) GSEA was performed to evaluate the role of NFATc2 in HCC using available data from TCGA. (**C**) Kaplan-Meier analysis of correlation between the NFATc2 expression and overall survival of HCC patients from TCGA (n=365). (**D** and **E**) Subcellular location of NFATc2 after FGF19 treatment: Huh-7 NCSCs were pre-treated with DMSO, BLU9931 (100 nM), 3-NC (20 µM), LY3214996 (2 µM), 3-NC (20 µM) + LY3214996 (2 µM), SKF96365 (5 µM), and FK506 (50 nM) for 2h, respectively; then treated with FGF19 (100 ng/ml) for 4h. (**D**) The levels of NFATc2 in cytosol and nucleus, and the phosphorylation level of NFATc2 were determined by WB, Lamin B served as a control of nuclear protein, and α-Tubulin was used as a control of cytoplasmic protein. (**E**) Subcellular location of NFATc2 (green) was detected by immunofluorescence (IF), and cell nucleus was labeled with DAPI (blue). Data are expressed as means ± SEM (n = 3). *p < 0.05, **p < 0.01, ***p < 0.001, *NS* represents no significant difference.

Recent studies indicate that NFATs dephosphorylation caused by SOCE promotes the stemness of CSCs *via* transcriptionally activating pluripotency transcription factors including Oct-4 and Sox2 27-29. Four isoforms of NFAT including NFATc1, NFATc2, NFATc3, and NFATc4 have been identified in human 30. To determine which isoforms of the NFATs are involved in FGF19-promoted self-renewal, we transfected vector-containing NFAT-response elements (NFAT-RE) into Huh-7 and RFP/PLC/5 NCSCs. As expected, FGF19 notably increased the luciferase activities of NFAT-RE, and knockdown of NFATc2 remarkably attenuated the NFAT-RE luciferase activities induced by FGF19 stimulation, which could not be shared by silencing other NFAT isoforms (Figure 5A and S6A-B).

Next, Gene Set Enrichment Analysis (GSEA) analyses of microarray data were obtained from The Cancer Genome Atlas (TCGA), which revealed the association between NFATc2 and pluripotency of stem cells pathways (Figure 5B). However, no significant associations between stem cell pluripotency and NFATc1, NFATc3 or NFATc4 were observed (Figure S7). Furthermore, Kaplan-Meier estimates revealed that high NFATc2 expression correlated with poor survival among HCC patients *via* microarray data obtained from TCGA database (Figure 5C).

Once SOCE influx increases intracellular Ca^2+^ concentration, which activates the calmodulin (CaM)-calcineurin (CaN) pathway. Subsequently, calcineurin mediates the dephosphorylation of NFATc2. Then the dephosphorylated NFATc2 translocates to the nucleus and upregulates transcription of its target genes 31, 32. We found that FGF19 significantly reduced the p-NFATc2 (Ser53) level, enhanced the expression of p-CaMKI I (Th286), and promoted the nuclear translocation of NFATc2, which were suppressed by BLU9931 (FGFR4 inhibitor), 3-NC (PLCγ inhibitor), LY3214996 (ERK1/2 inhibitor), SKF-96365 (SOCE inhibitor) and FK506 (calcineurin inhibitor) (Figure 5D). Moreover, immunofluorescence results showed that FGF19-triggered NFATc2 nuclear translocation could be blunted by BLU9931, SKF-96365 and FK506 (Figure 5E). These results suggest that SOCE-calcineurin axis is critical for NFATc2 nuclear accumulation in LCSCs.

**Figure 6:**
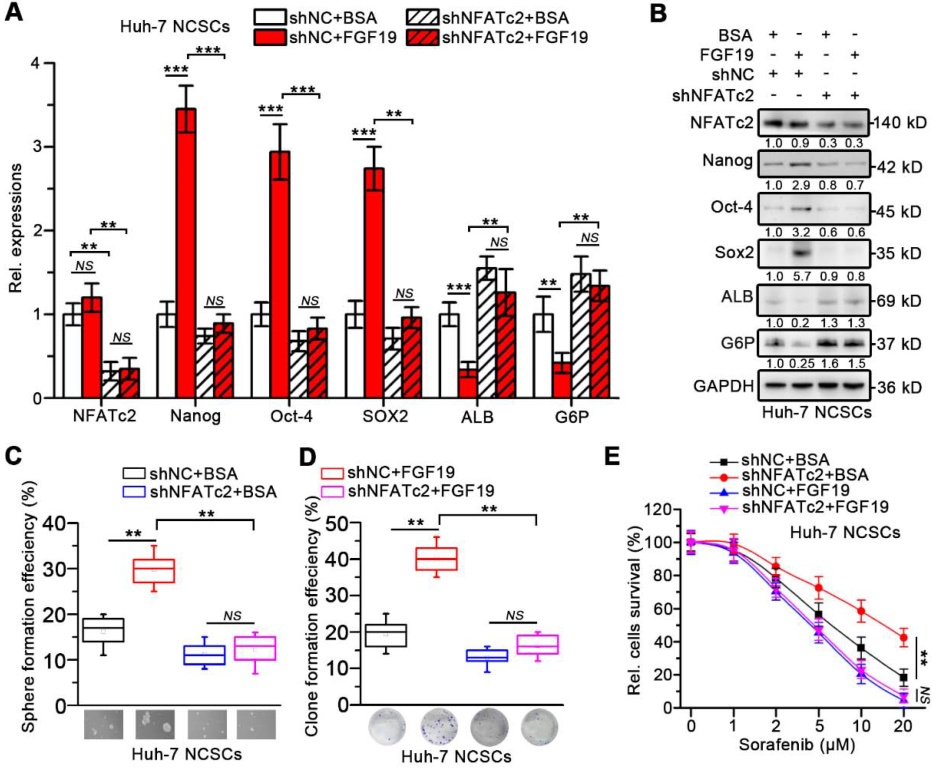
** Silencing NFATc2 attenuates FGF19-promoted self-renewal in non-LCSCs.** (**A-E**) The effects of silencing NFATc2 on FGF19-triggerd self-renewal in Huh-7 NCSCs were evaluated by (**A**) RT-qPCR and (**B**) WB measuring the expressions of Nanog, Oct-4, Sox2, ALB, and G6P, (**C**) sphere formation assay, (**D**) clonogenicity assay and (**E**) sorafenib resistance assay Data are expressed as means ± SEM (n = 3). *p < 0.05, **p < 0.01, ***p < 0.001, *NS* represents no significant difference.

In addition, loss of NFATc2 in FGF19-treated Huh-7 cells was associated with decreased expressions of stemness-related genes and increased expressions of mature hepatocyte markers (Figure 6A-B). FGF19 failed to promote the capability of sphere and clone formation, sorafenib resistance in NFATc2-deficient Huh-7 NCSCs (Figure 6C-E). These observations suggest that NFATc2 activation is pivotal for self-renewal promoted by FGF19.

### NFATc2 transcriptionally activate expression of FGF19

**Figure 7:**
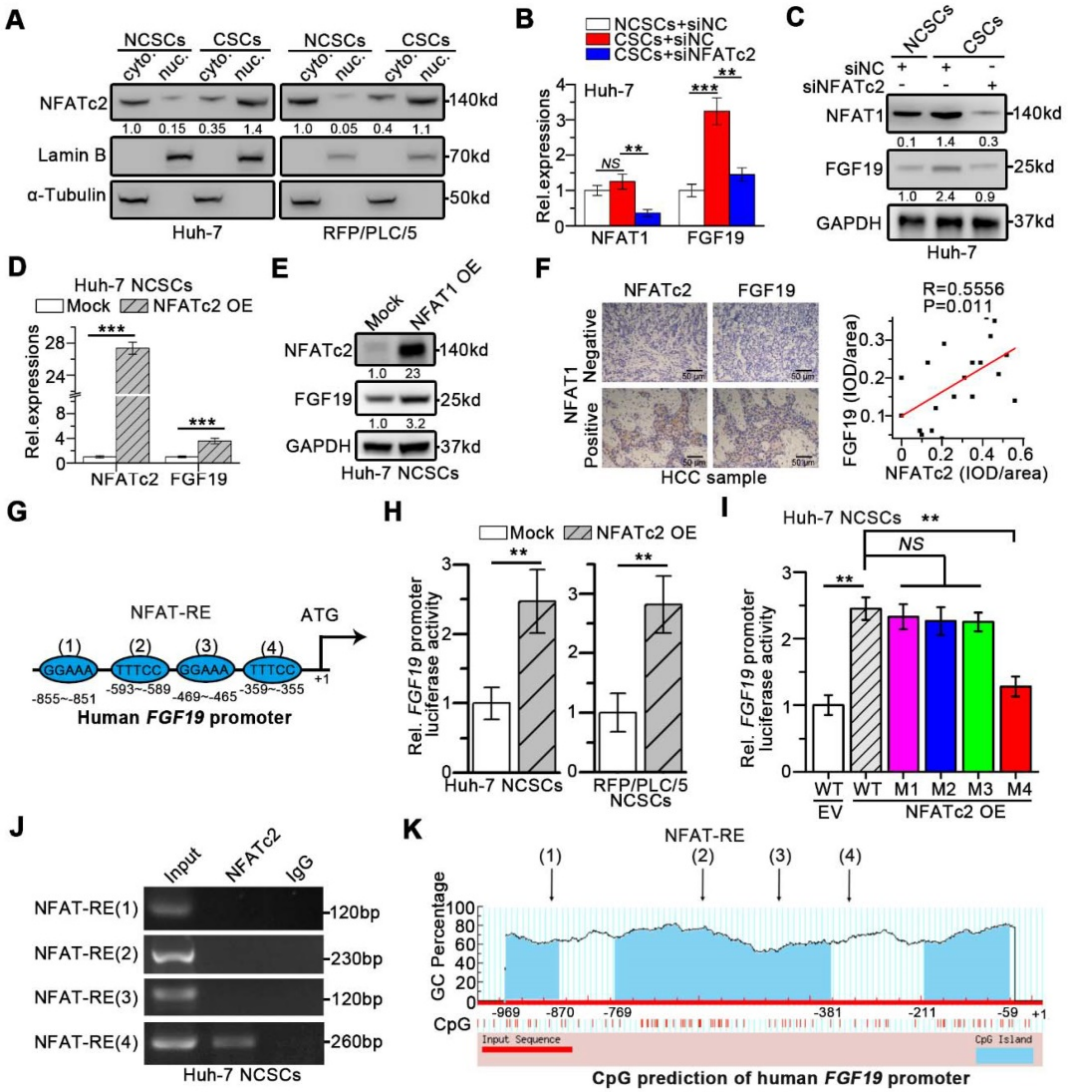
** NFATc2 transcriptionally increase FGF19 expression *via* binding* FGF19* promoter.** (**A**) The levels of NFATc2 in cytosol and nucleus from NCSCs and CSCs of HCC cells were determined by WB. NFATc2 and FGF19 mRNA (**B**) and protein (**C**) levels in NCSCs and CSCs of Huh-7 cells were transfected with siNC or si-NFATc2. RT-qPCR (**D**) and WB (**E**) to assess NFATc2 and FGF19 expressions in mock- and NFATc2 OE-Huh-7 NCSCs. (**F**) Representative micrographs (400×) of IHC analysis NFATc2 and FGF19 in 20 HCC samples, and analysis correlation between the IOD of NFATc2 and FGF19 against IgG. (**G**) Bioinformatics analysis predicted four NFAT-REs in the promoter of human *FGF19*. (**H**) Luciferase activity assay of *FGF19* promoter in Mock- and NFATc2 OE- NCSCs were determined. (**I**) Luciferase activities of wildtype FGF19 promoter (WT) and *FGF19* promoter containing single mutant NFAT-RE (M1 to 4) in Mock- or NFATc2 OE- Huh-7 NCSCs were measured. (**J**) ChIP assay of NFATc2 and *FGF19* promoter, representative agarose gel results showing recruitment of NFATc2 to the region containing the 4^th^ NFAT-RE, and IgG used as a negative control. (**K**) CpG island prediction results showed that there are 3 CpG islands in human *FGF19* promoter. Data are expressed as means ± SEM (n = 3). *p < 0.05, **p < 0.01, ***p < 0.001, *NS* represents no significant difference.

We next wondered whether NFATc2 could upregulate FGF19 thereby formed a positive circuit to promote the self-renewal of LCSCs. As expected, nuclear NFATc2 levels were markedly higher in LCSCs than in their corresponding NCSCs (Figure 7A). Knock-down of NFATc2 in LCSCs led to a dramatic reduction of FGF19 expression (Figure 7B-C). Consistently, NFATc2 OE enhanced the expressions of FGF19 in Huh-7 and RFP/PLC/5 NCSCs (Figure 7D-E). IHC results of HCC samples delineated a positive correlation between NFATc2 and FGF19 levels (Figure 7F).

Moreover, the *in silico* analysis revealed four specific NFAT-REs within the FGF19 promoter (Figure 7G). The reporter activity of *FGF19* promoter was heightened by NFATc2 OE in Huh-7 and RFP/PLC/5 NCSCs, whereas the specific mutation at the 4^th^ NFAT-RE attenuated the ability of NFATc2-enhanced FGF19 promoter activity (Figure 7H-I). Chromatin immunoprecipitation (ChIP) analysis showed that only DNA fragment containing the 4^th^ NFAT-RE could be amplified from the NFATc2-immunoprecipitated samples (Figure 7J). The CpG island prediction showed that the 4^th^ NFAT-RE was not in CpG islands region of *FGF19* promoter (Figure 7K). Together, these observations reveal that NFATc2 transcriptionally activates FGF19 in LCSCs *via* binding with the *FGF19* promoter at NFAT-RE.

### Targeting FGF19-NFATc2 signaling suppresses self-renewal of LCSCs

**Figure 8:**
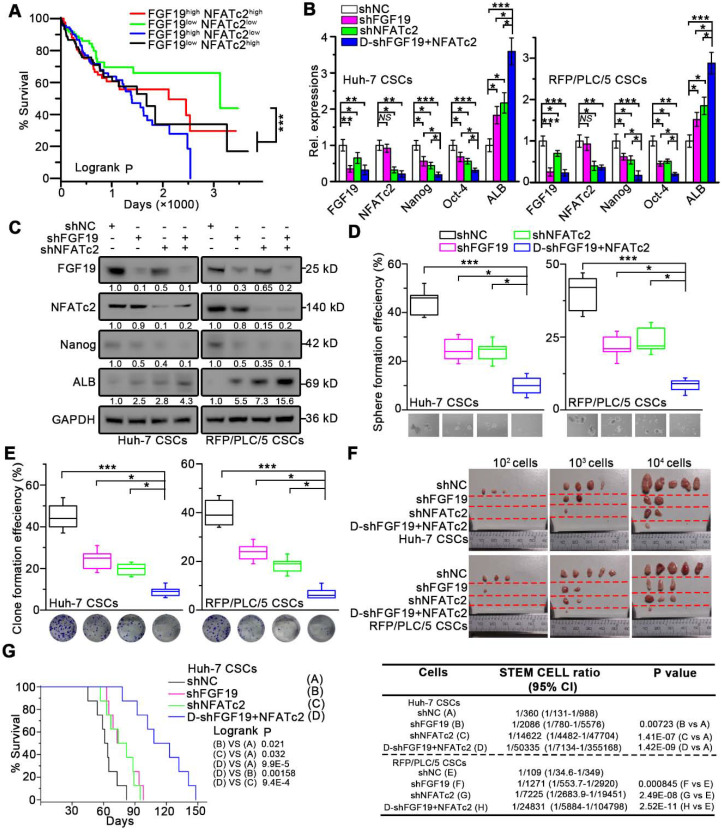
** Dual-knockdown of FGF19 and NFATc2 potently represses self-renewal of LCSCs.** (**A**) Kaplan-Meier analysis of correlation between the expression of FGF19 and NFATc2 with overall survival of HCC patients from TCGA (n = 365). (**B-F**) Huh-7 and RFP/PLC/5 CSCs were transfected with shNC, either shFGF19, or shNFATc2, shRNAs containing both shFGF19 and shNFATc2 (D-shFGF19 + NFATc2). (**B**) RT-PCR and (**C**) WB were applied to measure levels of FGF19, NFATc2, Nanog, Oct-4, and ALB. The effects of dual-silencing of FGF19 and NFATc2 on self-renewal features of LCSCs in Huh-7 and RFP/PLC/5 were assessed by (**D**) sphere formation assay, (**E**) clonogenicity assay, and (**F**) tumorigenic potential assays *in vivo*. (**G**) Kaplan-Meier survival curve of orthotopic liver tumor by intrahepatic implantation of Huh-7 LCSCs infected with transfected with shNC, either shFGF19, or shNFATc2, D-shFGF19 + NFATc2 (n = 8/group). Data are expressed as means ± SEM (n = 3). *p < 0.05, **p < 0.01, ***p < 0.001.

We next validated the potential clinical significance of targeting FGF19-NFATc2 signaling circuit. Kaplan-Meier estimates obtained from TCGA database revealed HCC patients with FGF19^low^ NFATc2^low^ showed significant longer overall survival, comparing to other three groups of HCC patients (Figure 8A). Thus, we respectively constructed FGF19 and NFATc2 dual-knockdown cell lines in LCSCs of Huh-7 and RFP/PLC/5 (Figure 8B-C). Compared with the control group, the absence of FGF19 or NFATc2 suppressed the self-renewal characteristics of LCSCs, including stemness-associated gene expressions (Figure 8B-C), clone and sphere formation (Figure 8D-E), and capability of tumor formation (Figure 8F). More importantly, the dual-knockdown of FGF19 and NFATc2 showed the lowest self-renewal activity compared to other 3 groups (shNC, shFGF19 and shNFATc2) (Figure 8B-F). Consistently, the data from orthotopic tumor model showed that animals implanted with Huh-7 LCSCs which dual knocked-down FGF19 and NFATc2 had longer survival than other 3 groups (Figure 8G). Furthermore, combined administration of BLU9931 and FK506 exerted stronger inhibitory effect on clone and sphere formation capability, tumor formation ability, and sorafenib resistance than individual inhibitor (BLU9931 or FK506)-treated groups (Figure S8A-D). These results demonstrate that targeting FGF19-NFATc2 signaling is a potential therapeutic approach to suppress the self-renewal of LCSCs.

## Discussion

Self-renewal is a critical characteristic by which CSCs drive tumorigenesis, cancer relapse and therapy-resistance 4, 5. High levels of FGF19 have been associated with poor outcome in HCC patients 10, 33. Here, our present study shows that FGF19 promotes the self-renewal of LCSCs *via* activating FGFR4/SOCE/NFATc2 pathway, in turn; NFATc2 mediates the transcriptional activation of FGF19. These findings indicate that FGF19/NFATc2 circuit is involved in maintaining the self-renewal of LCSCs, which may represent a potential target for cancer therapy.

Evidence showed that FGF2 (Basic FGF)/FGFR1-3-mediated signaling pathwaywas involved in maintaining CD44^High^/CD133^High^ CSCs in HCC 34. Besides, CD13^+^/CD166^-^ CSCs of Li-7 cell line exhibited higher mRNA levels of FGF3 and FGF4 35. It has been reported that FGF19 activates FGFR4 to mediate EMT, apoptosis resistance, sorafenib resistance, metabolism regulation and bile acid synthesis 14, 36. FGFR4 are significantly elevated in approximately one-third of HCC patients 8, and FGF19/FGFR4 axis possesses pro-tumor activities in multiple types of cancer including HCC, CRC, lung, breast and prostate cancer 36-40. FGF15, the murine orthologue of FGF19, also behaves as an enterohepatic hormone regulating bile acid synthesis *via* activating FGFR4 41.

Nowadays, the effect of FGF15/FGFR4 axis on hepatocarcinogenesis is controversial 25, 42. On one hand, FGF15 activates Mst1/2-SHP-Cyp7A1 signaling to suppress bile acid synthesis and suppresses hepatocarcinogenesis in the Mst1/2 deficiency mice, in which increased bile acid is an important cause of hepatocarcinogenesis 25. On the other hand, Fgf15^-^/^-^ mice displayed less and smaller tumors, and histological neoplastic lesions were also smaller than in Fgf15^+^/^+^ animals after DEN plus CCl4 administration, a model in which HCC developed via a pro-fibrotic and inflammatory pathway 42. We and other groups found that FGF19 expression is significantly higher in HCC compared to non-malignant liver 10, 13; high expression of FGF19 correlates with tumor progression and poorer prognosis of HCC 33. Metabolomic studies showed that content of bile acids in human HCC tumor tissues was significantly lower than that in adjacent normal tissues 43. These suggest that the upregulation of FGF19 in HCC is not due to bile acids, which is an important cause of hepatocarcinogenesis in Mst1/2 deficient mice. Moreover, we identified NFATc2 as a transcriptional factor of human FGF19 gene. Our results indicated that FGF19 promoted SOCE by activating PLCγ and ERK pathway, resulting in enhanced self-renewal characteristics in Huh-7 and RFP/PLC/5 cells. FGF19/SOCE/NFATc2 signaling circuit is oncogenetic and important for the self-renewal of LCSCs.

Recent study has shown that Ca^2+^ oscillations is critical for maintaining the stemness and survival of LCSCs, and SOCE is the major route of Ca^2+^ influx for HCC cells 17, 23. Several tumor-promoting growth factors, such as EGF, basic FGF (FGF2) and transforming growth factor beta (TGF-β), not only activate SOCE, but also enhance CSCs-like characteristics of cancer cells 44-48. It has been reported that FGF19 activates Wnt/β-catenin and PI3K/AKT/mTOR pathways in cancer cells 10, 39, which are related to both the regulation of SOCE and maintenance of CSC-like properties 49. However, the mechanism of FGF19 promoting Ca^2+^ signaling needs further exploration. Herein, we found that FGF19-promoted SOCE was depended on the activation of PLCγ and ERK1/2 pathways.

It has been reported that in cardiomyocytes, elevated intracellular Ca^2+^ activates calmodulin, which interacts with the kinase or the phosphatase 50 However, the effect of CaMKII on CaN-NFATs remains controversial 51, 52. In our study, both CaMKII and CaN were activated after FGF19 treatment. Higher level of phosphorylated CaMKII was observed in LCSCs compared with NCSCs. It's well known that SOCE promotes CaM-CaN pathway 31, 32. Consistently, we found that FK506, a specific inhibitor of CaN, significantly attenuated FGF19-triggered dephosphorylation of NFATc2 nuclear translocation, which demonstrated that CaN activation was necessary for FGF19-induced NFATc2 activation and nuclear translocation.

Accumulating evidence demonstrated that NFATs play an important role in tumorigenesis by regulating downstream target genes 28, 29, 53. For example, NFATc1 drives EMT and maintains pancreatic cancer cells in a CSC phenotype through Sox2-dependent transcription of EMT and stemness factors 29; NFATc3 plays an important role in the maintenance of CSC phenotype of oral/oropharyngeal squamous cell carcinomas (OSCCs) by promoting expression of Oct-4 28, 54. However, NFATc1 is frequently inactivated in HCC and functions as a tumor suppressor in liver carcinogenesis 55; NFATc3 inhibits hepatocarcinogenesis and HBV replication *via* promoting RIG-I-mediated interferon transcription 56. These reports imply that the role of NFAT isoforms may play different roles in distinct types of cancer.

In our present study, results of loss function assays suggest that NFATc2 plays an important role in regulating FGF19/FGFR4-promoted self-renewal, while silencing of NFATc1, c3 and c4 couldn't attenuate the NFAT-RE luciferase activities induced by FGF19 stimulation. GSEA analysis revealed that there was no significant association between NFATc1, NFATc3 or NFATc4 and function of stem cell pluripotency. Taken together, our study indicates that FGF19-FGFR4-NFATc2 pathway is important in regulating HCC stemness. To our knowledge, our finding is the first report showing that the expression of NFATc2 is correlated with the stemness and self-renewal of HCC, as well as poor outcome among HCC patients.

NFATc2 protein is a Ca^2+^-regulated transcription factor that controls gene expression in many cell types. NFATc2 is phosphorylated (*e.g.* Ser53, Ser 326) and resides in the cytoplasm of resting cells; when cells are stimulated by rising intracellular Ca^2+^ level, NFATc2 is dephosphorylated by the Ca^2+^/calmodulin-dependent phosphatase calcineurin and translocates to the nucleus to activate target gene expression 31, 32. We found that SOCE activated-NFATc2 nuclear accumulation was required for FGF19-dependent self-renewal, in turn, NFATc2 transcriptionally activated the expression of FGF19. Finally, dual-knockdown of FGF19 and NFATc2 synergistically blunted the self-renewal of LCSCs and prolonged survival of corresponding xenograft tumor-bearing mice.

To date, the specific inhibitors for FGFR4 (*e.g*. BLU9931 and BLU554), SOCE (*e.g*. 2-APB and SKF96365) and calcineurin (*e.g*. cyclosporine A and FK506) are available for clinical or preclinical studies and display excellent anti-tumor activities 17, 57. Our findings reveal the underlying mechanism by which SOCE is involved in the self-renewal of LCSCs, and highlight the importance of FGF19-NFATc2 signaling circuit as a potential therapeutic target to eliminate LCSCs and improve the efficacy of HCC therapy in clinics.

## Materials and methods

### Human samples

20 pairs of HCC tissues were obtained from patients at the Department of Hepatobiliary Surgery, Xinqiao Hospital (Chongqing, China). The use of clinical specimens in this study was approved by Ethical Review Board of the Xinqiao Hospital ethics committee, and informed written consent was obtained from all participants.

### Cell culture and spheroid culture

RFP/PLC/5 and HEK293T cells were obtained from ATCC (MD, USA). Huh-7 and MHCC97H were obtained from the Cell Bank of Type Culture Collection of Chinese Academy of Sciences (Shanghai, China). All cells lines were authenticated and tested for mycoplasma, and maintained according to the manufacturer's instructions. For spheroid culture, cells were plated into ultra-low attached dishes (Nest, Wuxi, China). Cells were cultured in DMEM/F12 media (Thermo Fisher, Grand Island, NY, USA) with penicillin, streptomycin, B27 supplement (Gibco), 20 ng/mL of epidermal growth factor (EGF, PeproTech, Rocky Hill, NJ, USA), 20 ng/mL of basic FGF (bFGF, PeproTech), and 5 U/mL of insulin (PeproTech). Equal fresh media was added every 3 days. For passaging, spheres were collected by gentle centrifugation after culturing for 2 weeks and dissociated into single cells with StemPro Accutase Cell Dissociation Reagent (Gibco).

### Lentiviral and siRNA

The recombinant lentiviral plasmids containing human FGF19 or NFATc2, and shRNA-targeting FGF19 or NFATc2 were purchased from GeneCopoeia (Rockville, MD, USA). Lentivirus was produced in HEK293T cells according to the instruction manual of Lenti-Pac™ HIV Expression Packaging Kit (GeneCopoeia). Supernatants containing viral were harvested at 72 h post-transfection, passed through a 0.45 µm polyethersulfone low protein-binding filter, diluted 1:1 (v/v) with fresh medium containing polybrene (7.5 mg/ml). For generating FGF19-deficient LCSCs, the spheroids of LCSCs were dissociated single cells, and then infected with lentiviral carrying red fluorescence protein (RFP)-sh-FGF19. Three days after infection, the RFP positive cells were sorted with flow cytometry. For generating NFATc2-deficient LCSCs, the spheroids of LCSCs were dissociated to single cells, and then infected with lentiviral carrying green fluorescence protein (GFP)-sh-NFATc2. Three days after infection, the GFP positive cells were sorted *via* flow cytometry. Over-expression or knockdown efficiency of FGF19 and NFATc2 were evaluated by RT-qPCR and Western-blotting. The siRNAs targeting human NFATc2 (stB0007239A-1-5), NFATc1 (stB0014378A-1-5), NFATc4 (stB0007242A-1-5) and NFATc3 (stB0007241A-1-5) were obtained from RiboBio Inc. (Guangzhou, China). Cells were transfected by Lipofectamine^TM^2000 (Thermo Fisher) according to the manufacturer's instructions.

### CRISPR/Cas9 targeted deletion of *FGFR4*

To knock out *FGFR4* gene in Huh-7 and RFP/PLC/5 NCSCs, we designed single guided RNA (sgRNA) sequences (Forward 5′-CAC CGT GTG CGG CTG TGC TGT GGG C -3′; Reverse: 5′-AAA CGC CCA CAG CAC AGC CGC ACA C-3′) for human *FGFR4* gene and cloned the targeting sequences into the lentiCRISPR v2 vector (Addgene, Watertown, MA, USA). Lentivirus for *FGFR4* sgRNA and control vector were produced in HEK293T cells using lenti-packaging vector according to standard methods. Huh-7 and RFP/PLC/5 NCSCs were then infected with the lentivirus for 48 h and selected with puromycin (2.5 μg/ml) for 10 days, then the monoclonal NCSCs were established. FGFR4 deletion in individual monoclonal cells was further analyzed by DNA sequencing and WB.

### Sorafenib resistance assay

Sorafenib resistance of cells was determined by Cell Counting Kit-8 (CCK-8 Kit, Dojindo, Kumamoto, Japan) according to the manufacturer's instructions. Briefly, both adherent cells (NCSCs) and spheroids (CSCs) were dissociated into single cells, then were seeded in a 96-well plate (2×10^3^ cells/well), and incubated in complete culture medium (2D culture) containing various concentrations of sorafenib for 48 h. CCK-8 reagent was added to each cell-containing well. After incubation for 2 h, the absorbance was quantified at 450 nm using Varioskan Flash (Thermo Fisher Scientific), none-cell wells as blank control.

### Sphere formation assay

100-cells were plated into ultra-low attachable 12-well plates (Nest), cells were cultured in DMEM/F12 media with penicillin, streptomycin, B27 supplement, 20 ng/mL of EGF, 20 ng/mL of bFGF, and 5 U/mL of insulin. Equal fresh media was added every 3 days. After culturing for 2 weeks, spheres with diameter > 75 μm were counted.

### Clone formation assay

100-single cells were seeded in 12-well plates with DMEM medium containing 10% FBS. After culturing for 2 weeks, cell clones were fixed by 4% paraformaldehyde (Beyotime, Beijing, China) and dyed with crystal violet (Beyotime). Clone (>50 cells) numbers were assessed by microscope.

### Animal studies

5-week-old male BALB/c-nude mice (Vital River, Beijing, China) were maintained in pathogen-free conditions. For tumor formation assay, the cells were serially diluted (10^4^, 10^3^ and 10^2^ cells) in serum-free medium and were mixed with Matrigel (BD Biosciences, CA) at 1:1. The mixtures were subcutaneously injected into nude mice. Tumor formation was monitored regularly after subcutaneous injection. All mice were sacrificed by cervical dislocation on 5 weeks after injection. All animal experiments met the ethical principles and requirements of our committee and comply with the Declaration of Helsinki. The ratios of stem cells were calculated by ELDA (extreme limiting dilution analysis) with online software (http://bioinf.wehi.edu.au/software/elda/). For survival probability, 10^5^ cells in serum-free medium and were mixed with Matrigel were injected into the livers of BALB/c-nude mice. Survival was monitored in all animals every day. Overall survival probabilities of tumor-bearing mice were evaluated using the Kaplan-Meier method.

### Gene expression

Total RNA was extracted from cells using RNAiso Plus (TAKARA, Japan) and reverse transcribed using PrimeScript™ RT reagent Kit with gDNA Eraser (TAKARA) according to the manufacturer's instructions. mRNA expression was assessed by RT-quantitative PCR (qPCR) using TB Green® Premix Ex Taq™ II (TAKARA) on BioRad CFX386 (Bio-Rad, CA) with 40 cycles at 95 °C for 10 s, 59 °C for 20 s and 72 °C for 30 s. Gene expression levels were analyzed using the delta Ct method and normalized by subtracting that of glyceraldehyde-3-phosphate dehydrogenase (GAPDH). The gene-specific primers used in RT-qPCR experiments were listed in Table S1.

### Western blotting

Whole cell protein lysates were prepared by direct lysis in RIPA buffer with PMSF (Beyotime) and phosphatase inhibitors (Cwbiotech, Beijing, China). Proteins were quantified using BCA Protein Assay Kit (Beyotime). Samples were then separated by 4-12% Bis-Tris PAGE electrophoresis and transferred to PVDF membrane for detection. Western blots were probed overnight at 4 °C, with specific primary antibodies in Tris-Buffered Saline Tween-20 (TBST) containing 5% skim milk. After washed for 3 times with TBST, the membranes were incubated for 1 h at room temperature with a respective IgG-HRP labeled second antibody (1:5000, Zhongshan Goldenbridge, Beijing, China) in TBST containing 5% skim milk. Antigens were revealed using a chemiluminescence assay (Pierce, Rockford, USA). GAPDH was used as a control of total protein, Lamin B1 served as a control of nuclear protein, and α-tubulin was used as a control of cytoplasmic protein. Quantification of bands was achieved by densitometry using the Image J software. The antibodies used in WB and IHC analysis are listed in Table S2.

### Immunofluorescence

Immunofluorescence (IF) staining of NFATc2 was performed on tumor cells cultured on chamber slides (Thermo Fisher). The primary antibody was rabbit anti-human NFATc2 (CST, 5861), and secondary antibody were Anti-rat IgG (H+L), (Alexa Fluor® 555 Conjugate) (CST, 4417). Cells were fixed in ice-cold acetone/methanol (1:1) and stained with primary antibody (1:100), and secondary antibodies (1:1000). Cells were counterstained with DAPI and visualized with a fluorescent confocal microscope.

### Immunohistochemistry

The tissue specimens were placed for at least 24 h in 10% neutral-buffered formaldehyde immediately after removed from the mice. Hematoxylin and eosin (H&E), FGF19 (1:100 dilution), NFATc2 (1:100 dilution), Nanog (1:50 dilution), Oct-4 (1:100 dilution) and ALB (1:50 dilution) staining, along with IgG as a negative control, were performed on 4 mm sections. The mean density (IOD/area) of FGF19, NFATc2, Nanog and Oct-4 were detected in different positive areas of liver cancer specimens using ImageJ software. The antibodies used in IHC analysis are listed in Table S2.

### Calcium imaging

Calcium imaging was carried out as previously described 21, 58. Briefly, cells were placed on coverslips coated with poly-D-lysine. Intracellular Ca^2+^ was monitored using the fluorescent Ca^2+^ indicator Fura 2-AM according to the manufacture's instruction. Images were collected at 6-second intervals. Measurements of intracellular Ca^2+^ concentration ([Ca^2+^]_i_) of single cells were performed using an inverted fluorescence microscope (Nikon, Japan). The standard extracellular solution contained (mM): 140 NaCl, 5 KCl, 2 CaCl_2_, 1 MgCl_2_·6H_2_O, 10 HEPES, 10 Glucose, pH 7.4. Ca^2+^-free extracellular solution was prepared by replacing CaCl_2_ with equimolar amounts of MgCl_2_ and 0.5 mM EGTA was added. After loading, cells were washed three times in the above solution and then left for 15 min to allow for further de-esterification. Background fluorescence signals were collected at the same rate for the same wavelengths (340 and 380 nm) and were subtracted from the corresponding fluorescence images. The results (∆F/F0) were expressed as ratios of fluorescence signals measured at 340 nm to that at 380 nm during a response divided by the ratio obtained in resting conditions (that is, before the addition of an agent). ∆F/F0 was used to assess the amplitude of [Ca^2+^]i in these cells.

### ELISA

FGF19 concentration in supernatants were measured by using the Human FGF19 Quantikine ELISA kit (R&D Systems, MN, USA) according to the manufacturer's protocols. Secreted FGF19 protein levels were read at 450 nm within 30 min.

### STIM1 oligomerization assay

The CDS of STIM1 was cloned into pCMV-C-mCherry vector (Beyotime, D2628), and the STIM1-mcherry chimeric vector was transfected into Huh-7 NCSCs with Lipofectamine 2000. After treatment with recombinant FGF19 protein and inhibitors for 2 h, and cell images were photographed using a fluorescence microscope (Leica, Wetzlar, Germany). STIM1 puncta was counted using ImageJ software.

### Luciferase activity assay

pGL4.30 [luc2P/NFAT-RE/Hygrol] vector containing NFAT response elements were purchase from Promega. The promoter region (-1005 ~ +1 from the transcription starting site) was synthesized by GenScript co., LTD (Nanjing, China) and subcloned into pGL3-basic vector (Promega, WI, USA); and the 4 classical NFAT core motif sequence 5'-TTTCC was mutated to 5'-TTTAA, respectively. To examine the *FGF19* promoter activity, the mock or NFATc2 OE cells was transfected with 1μg of reporter vectors and 20 ng of pSV-Renilla expression vector. Luciferase and renilla activities were measured using the dual-luciferase reporter system kit (Promega), and the luciferase activity was normalized with renilla activity. The results are expressed as the averages of the ratios of the reporter activities from triplicate experiments.

### Chromatin immunoprecipitation PCR

Chromatin immunoprecipitation (ChIP) assays were performed using a SimpleChIP® Plus Enzymatic Chromatin IP Kit (CST, 9005) with ChIP-validated anti-NFATc2 (CST, 5861). Briefly, chromatin from cells was cross-linked with 1% formaldehyde for 10 min at room temperature, then digested to length of approximately 150-900 bp for 20 min at 37 °C, and immunoprecipitated with NFATc2 antibody and IgG (CST, 3900). The four pairs of ChIP-PCR primers were designed to amplify the region containing each NFAT-RE site at the* FGF19* promoter (Table S3). Each purified DNA was amplified by using ChIP-PCR products were analyzed by agarose gel electrophoresis.

### Chemicals and recombinant proteins

SKF-96365 (HY-100001), FK506 (HY-13756), BLU9931 (HY-12823), LY3214996 (HY-101494), 3-NC (HY-111919) and Puromycin (HY-B1743) were obtained from MedChemExpres (Monmouth Junction, NJ, USA). Lipofectamine 2000 (11668027), and Fura 2-AM (F1221) were obtained from Thermo Fisher Scientific (Waltham, MA, USA). Dimethyl sulfoxide (DMSO, 34869) and were purchased from Sigma-Aldrich (St. Louis, MO, USA). Recombinant FGF19 (100-32), EGF (100-15), and bFGF (100-18B) were obtained from PeproTech Inc.

### Statistical analysis

Pearson's correlation coefficient was used to determine the correlation between FGF19 and NFATc2 expressions in HCC samples examined by IHC (n=20). The LinkedOmics database was used to study genes that were correlated with NFATc1-4 59. GSEA was conducted for functional annotation with KEGG pathway enrichment analyses by using the open access WebGestalt tool 60. Overall survival of patients with HCC was evaluated using the Kaplan-Meier method, data were obtained from TCGA (n = 365), and the differences in survival curves were analyzed using the log-rank test. When two groups were compared, the Student's t test (unpaired) was used. Statistical analysis was performed using the statistical program Origin 9.1 (OriginLab, Northampton, MA, USA). All data are presented as mean ± SEM and were analyzed by Student's t test or one-way ANOVA. P values < 0.05 were considered statistically significant.

## Supplementary Material

Supplementary figures and tables.

## Abbreviations

ALB: albumin
ALDH1: aldehyde dehydrogenase 1
Ca^2+^: calcium
CaM: calmodulin
CaN: calcineurin
CaMKII: Ca^2+^/calmodulin dependent protein kinase II
ChIP: chromatin immunoprecipitation
CsA: cyclosporine A
EGF: epidermal growth factor
EMT: epithelial-mesenchymal transition
FGF: Fibroblast growth factor
G6P: glucose-6-phosphatase
GSEA: Gene Set Enrichment Analysis
HCC: hepatocellular carcinoma
IHC: immunohistochemistry
LCSCs: liver cancer stem cells
NCSCs: None-CSCs
NFAT: nuclear factors of activated T cells
NFAT-RE: NFAT-response elements
OCT4: octamer-binding protein 4
SOCE: store-operated Ca^2+^ entry
SOX2: SRY-box transcription factor 2
STIM1: stromal interaction molecule 1
TCGA: The Cancer Genome Atlas
TGF-β: transforming growth factor beta
