# Supplementary material for: FGF19/SOCE/NFATc2 signaling circuit facilitates the self-renewal of liver cancer stem cells
Source: Theranostics. 2021 Mar 5;11(10):5045–60. doi: 10.7150/thno.56369 (PMC7978301; doi:10.7150/thno.56369)
Supplement: Supplementary file 1 — Supplementary figures and tables. [file thnov11p5045s1.pdf]

Supplementary information

Number of supplementary figures = **8**, number of supplementary tables = **3**.

Supplementary figures

Figure S1

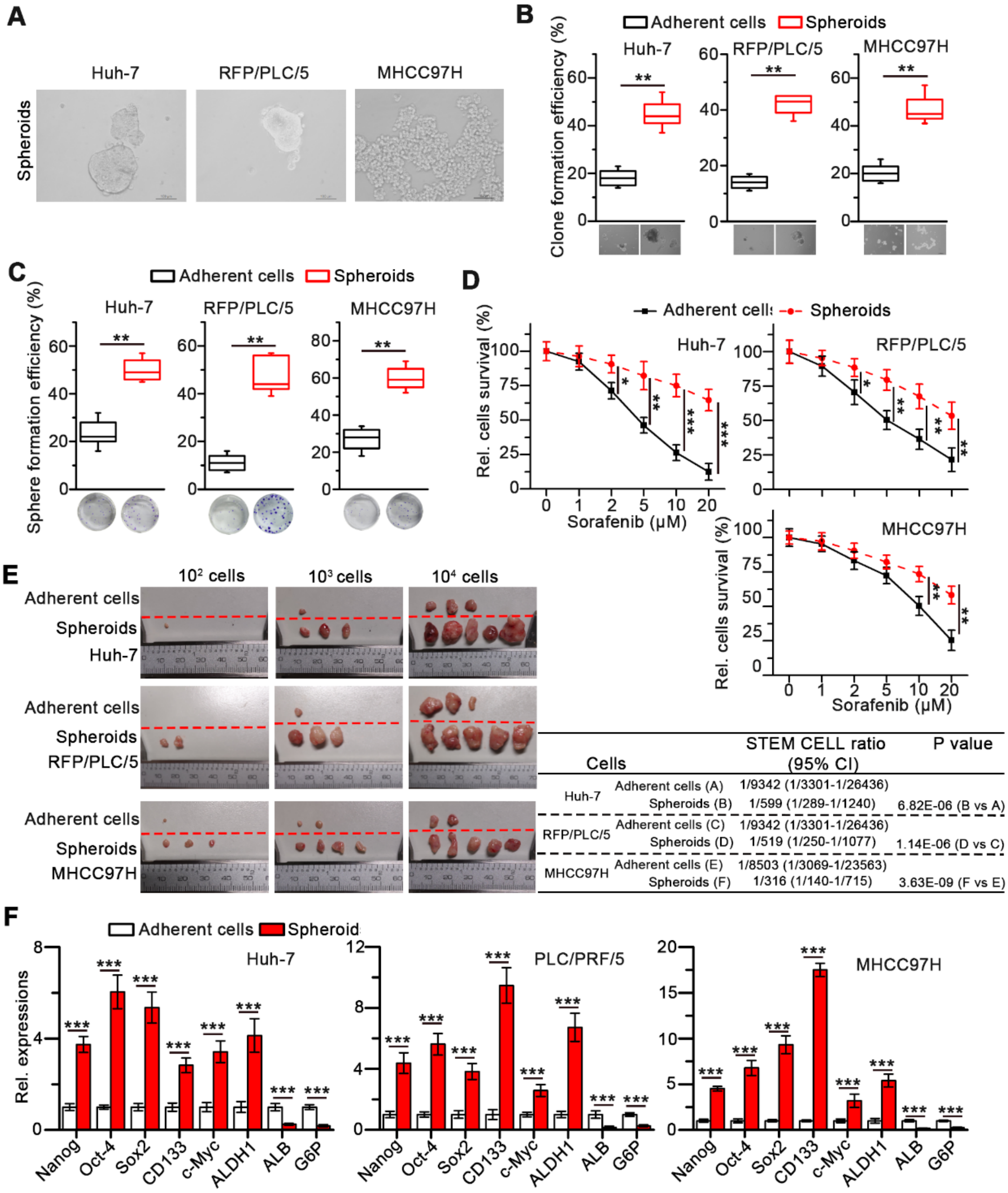

Figure S1. Establishment of LCSCs model by serum-free suspension culture. (**A**) Morphology of spheroids derived from Huh-7, RFP/PLC/5 and MHCC97H cells are shown (400×). (**B**-**E**) The self-renewal features in spheroids and their parent cells of

Huh7, RFP/PLC/5 and MHCC97H were assessed by (**B**) sphere formation assay, (**C**) clonogenicity assay, (**D**) sorafenib resistance assay and, and (**E**) tumorigenic potential assays *in vivo*. (**F**) RT-PCR was applied to measure mRNA levels of self-renew related genes (including *NANOG*, *OCT4*, *SOX2*, *CD133*, *c-MYC* and *ALDH1*), and the mature hepatocyte markers (including *ALB* and *G6P*) in spheroids and their parent cells of Huh7, RFP/PLC/5 and MHCC97H. Data are expressed as means ± SEM (n = 3). $^{*}p < 0.05$, $^{**}p < 0.01$, $^{***}p < 0.001$.

Figure S2

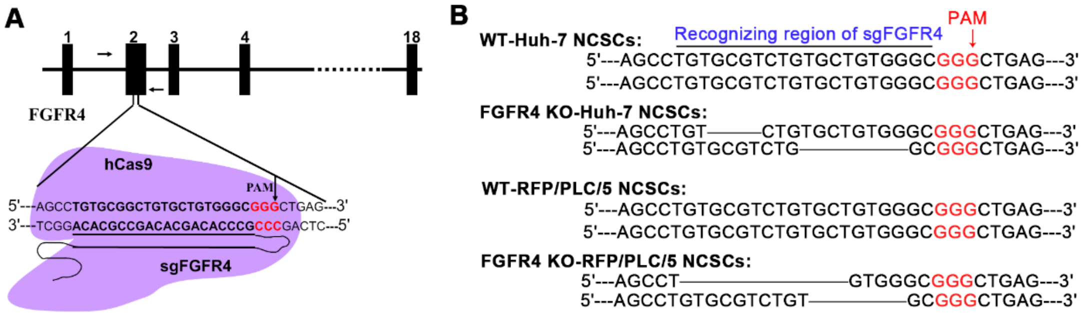

Figure S2. Knockout of FGFR4 using CRISPR/Cas9 in Huh7 and RFP/PLC/5. (**A**) Diagram depicts that the nuclease hCas9 recruited by a single guided RNA (sgRNA) specifically recognizing a region spanning the *FGFR4* codon (sgFGFR4) cleaves the *FGFR4* gene. The vertical arrow showed the potential cleavage site, PAM: protospacer adjacent motif. (**B**) Sequences in the Cas-9-edited-genome region in monoclones of *FGFR4* knockout (KO)-Huh7 and RFP/PLC/5 NCSCs by DNA sequencing, WT: wildtype. Data are expressed as means ± SEM (n = 3).

Figure S3

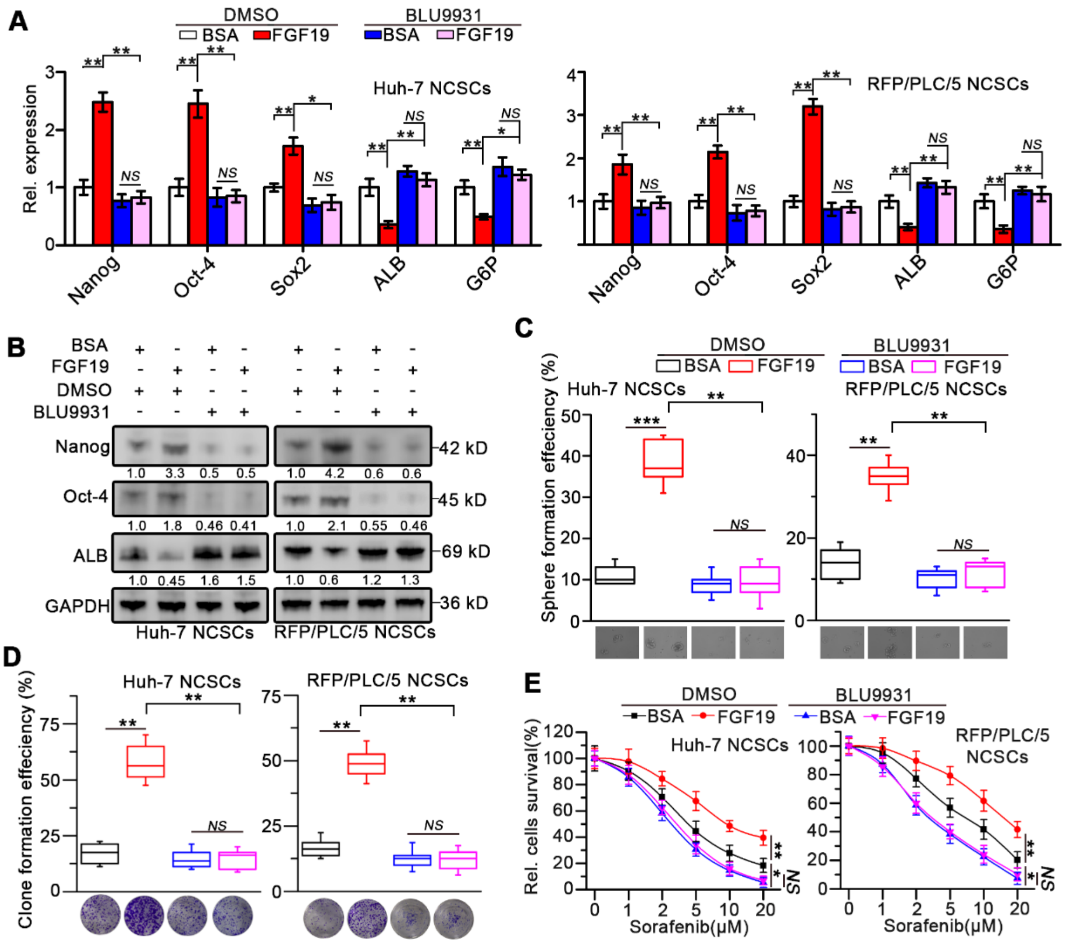

Figure S3. BLU9931 represses FGF19-induced self-renewal characteristics in Huh-7 NCSCs. (**A-D**) The effects of administration of BLU9931 on FGF19 (100 ng/ml)-triggerd self-renewal in Huh-7 NCSCs were evaluated by (**A**) RT-qPCR and (**B**) WB measuring the expressions of Naong, Oct-4, Sox2, ALB, and G6P, (**C**) sphere formation assay, (**D**) clonogenicity assay, and (**E**) sorafenib resistance assay. Data are expressed as means ± SEM (n = 3). $^*p < 0.05$, $^{**}p < 0.01$, $^{***}p < 0.001$, *NS* represents no significant difference.

Figure S4

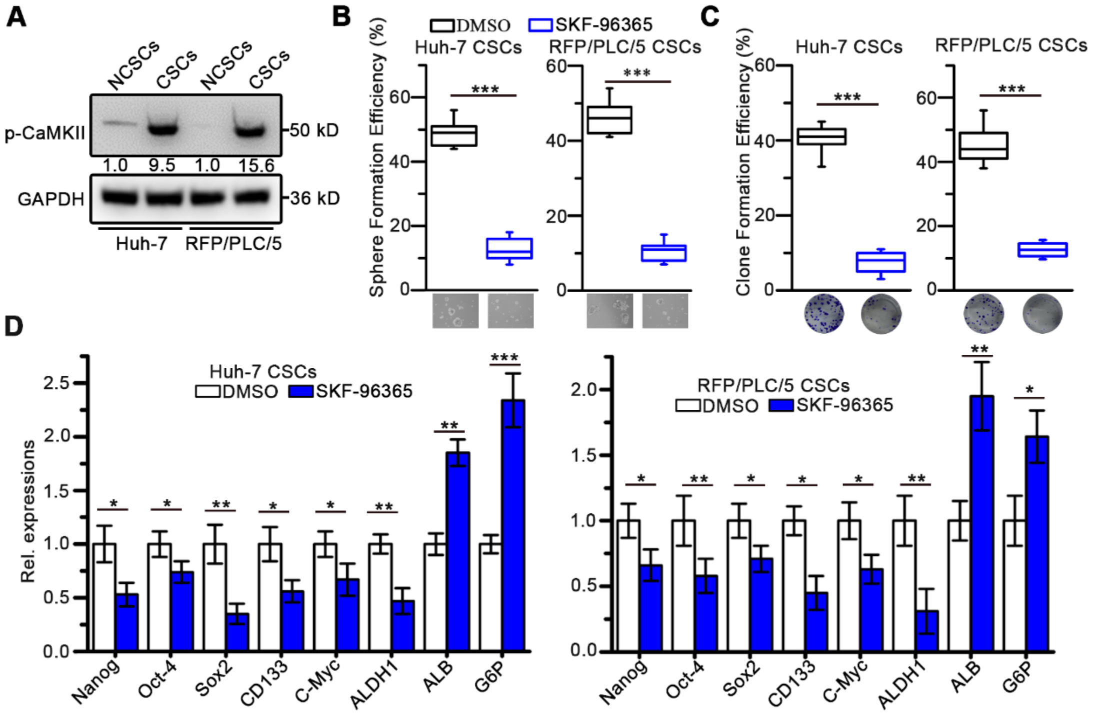

Figure S4 SOCE is essential for maintaining self-renew of LCSCs. (**A**) The expressions of p-CaMKII in CSCs and corresponding NCSCs of Huh7 and RFP/PLC/5 were measured by WB. The effects of SKF-96365 (5 μM) on self-renewal features of CSCs in Huh7 and RFP/PLC/5 were assessed by (**B**) sphere formation assay and (**C**) clonogenicity assay. (**D**) RT-PCR was applied to measure mRNA levels of self-renew related genes (including *NANOG*, *OCT4*, *SOX2*, *CD133*, *c-MYC* and *ALDH1*), and the mature hepatocyte markers (including *ALB* and *G6P*) in CSCs of Huh7 and RFP/PLC/5 treated with DMSO or SKF-96365 (5 μM) for 24 h. Data are expressed as means ± SEM (n = 3). *p < 0.05, **p < 0.01, ***p < 0.001.

Figure S5

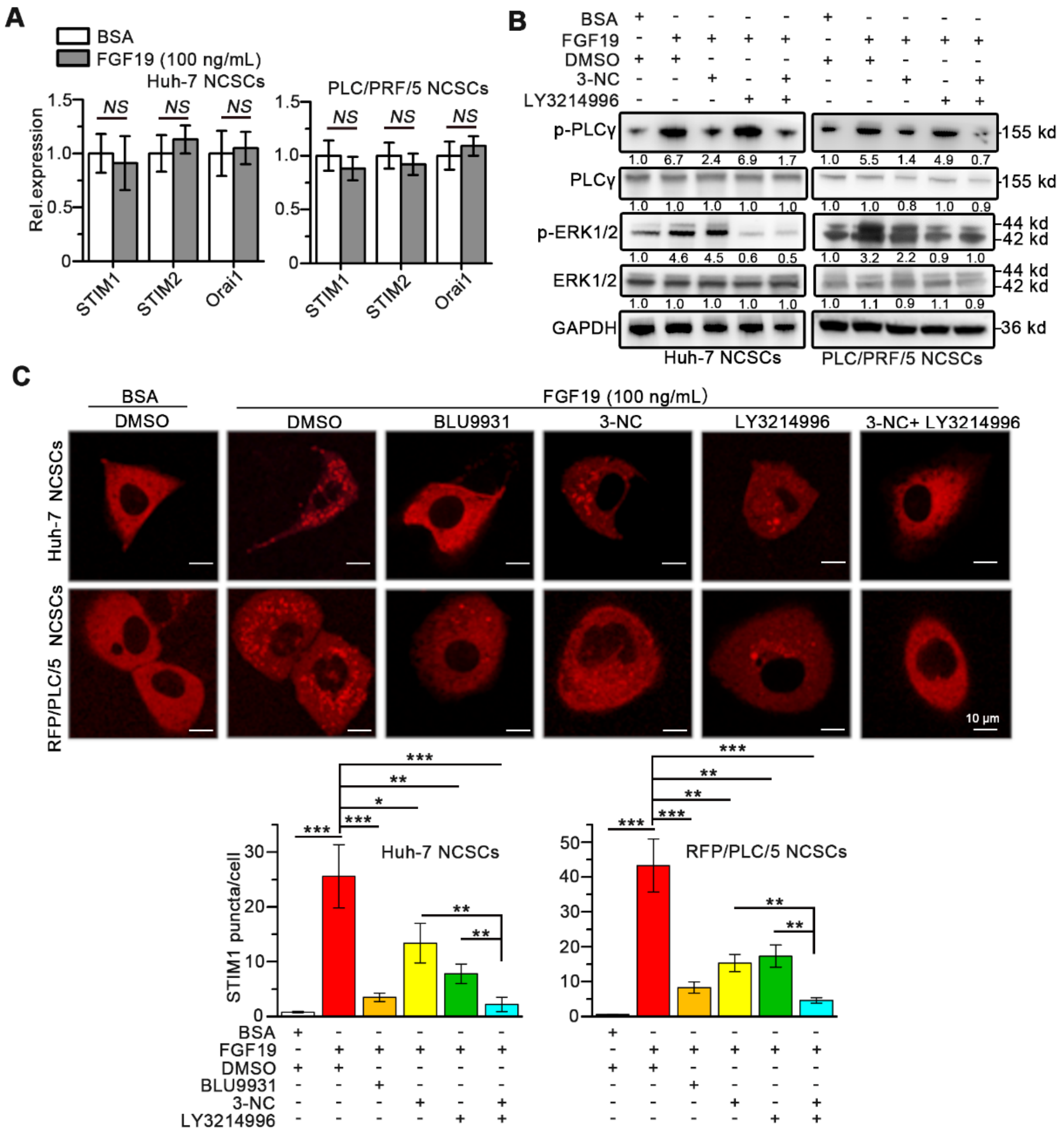

Figure S5 FGF19 significantly promotes aggregation of STIM1. (**A**) mRNA levels of STIM1, STIM2 and Orail1 were examined by RT-qPCR in FGF19 (100 ng/ml) treated- or BSA (100 ng/ml) treated Huh-7 and RFP/PLC/5 NCSCs for 4h. (**B**) Huh-7 and RFP/PLC/5 NCSCs were pre-treated with DMSO, BLU9931 (100 nM), 3-NC (20 μM), LY3214996 (2 μM), 3-NC (20 μM) + LY3214996 (2 μM), respectively; then disposed with FGF19 (100 ng/ml) for 4h. WB was used to measure the levels of p-PLCγ, PLCγ, p-ERK1/2 and ERK1/2, and GAPDH served as the control. (**C**) Huh-7 and RFP/PLC/5 NCSCs were transfected with STIM1-mcherry fusion recombinant plasmid, then were pre-treated with DMSO, BLU9931 (100 nM), 3-NC (20 μM), LY3214996 (2 μM), 3-NC (20 μM) + LY3214996 (2 μM), ~~SKF 96365 (5 μM),~~ and FK506 (50 nM) for 2h, respectively, and disposed with FGF19 (100 ng/ml) for 4h.

Cells were visualized under laser confocal microscopy (Nikon, Japan) to evaluate STIM1 multimerization. Data are expressed as means ± SEM (n =3). $*p < 0.05$, $**p < 0.01$, $***p < 0.001$, *NS* represents no significant difference.

Figure S6

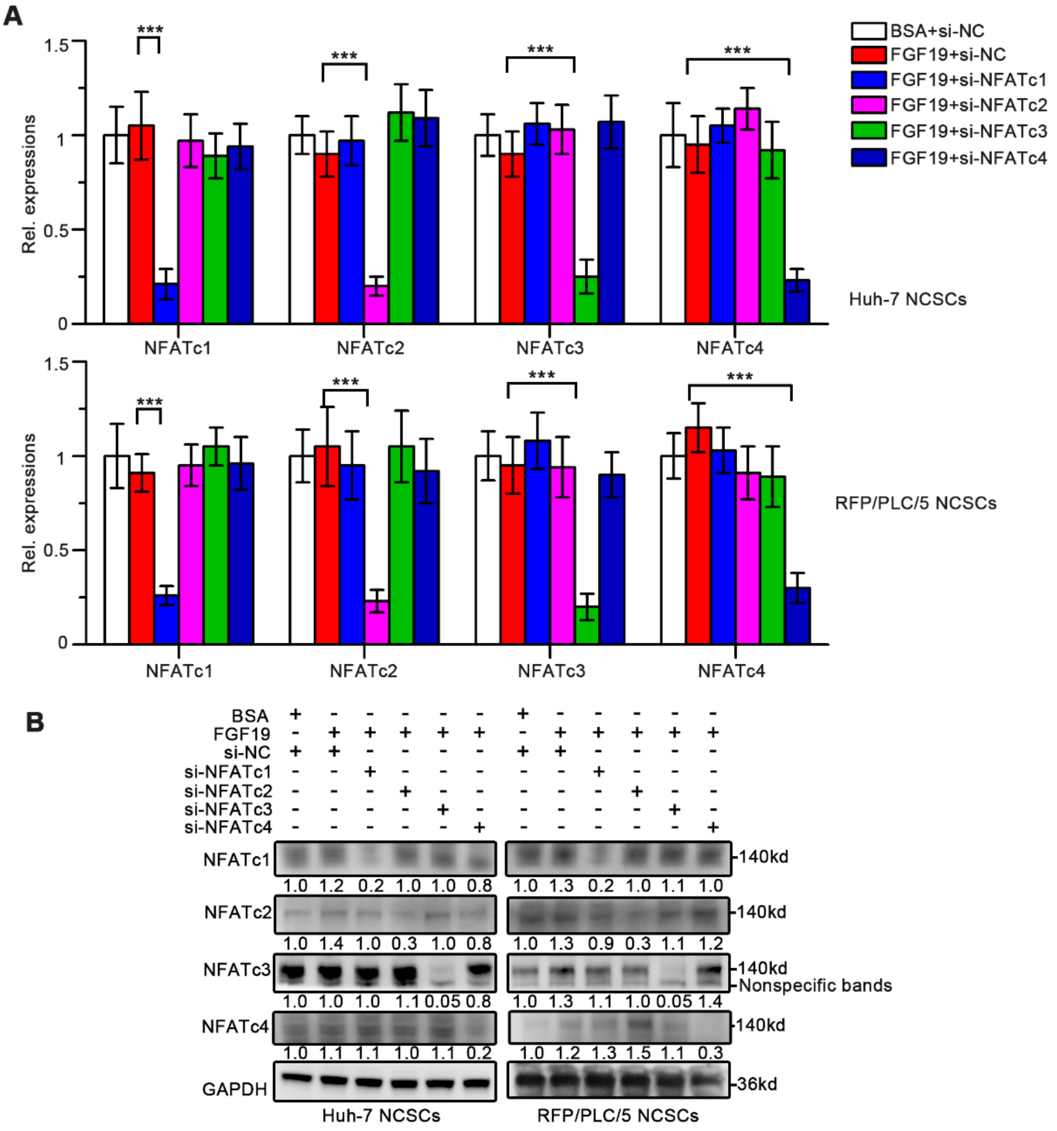

Figure S6. Silencing NFATc1-4 genes by siRNA. After transfection with si-NC, si-NFATc1, NFATc2, NFATc3 or NFATc4 for 24h, respectively; (**A**) mRNA levels of NFATc1, NFATc2, NFATc3 and NFATc4 were examined by RT-qPCR in FGF19 (100 ng/ml) treated-Huh-7 and RFP/PLC/5 NCSCs for 4h. (**B**) Protein levels of NFATc1, NFATc2, NFATc3 and NFATc4 were detected by WB. Data are expressed as means ± SEM (n = 3). ***$p < 0.001$.

Figure S7

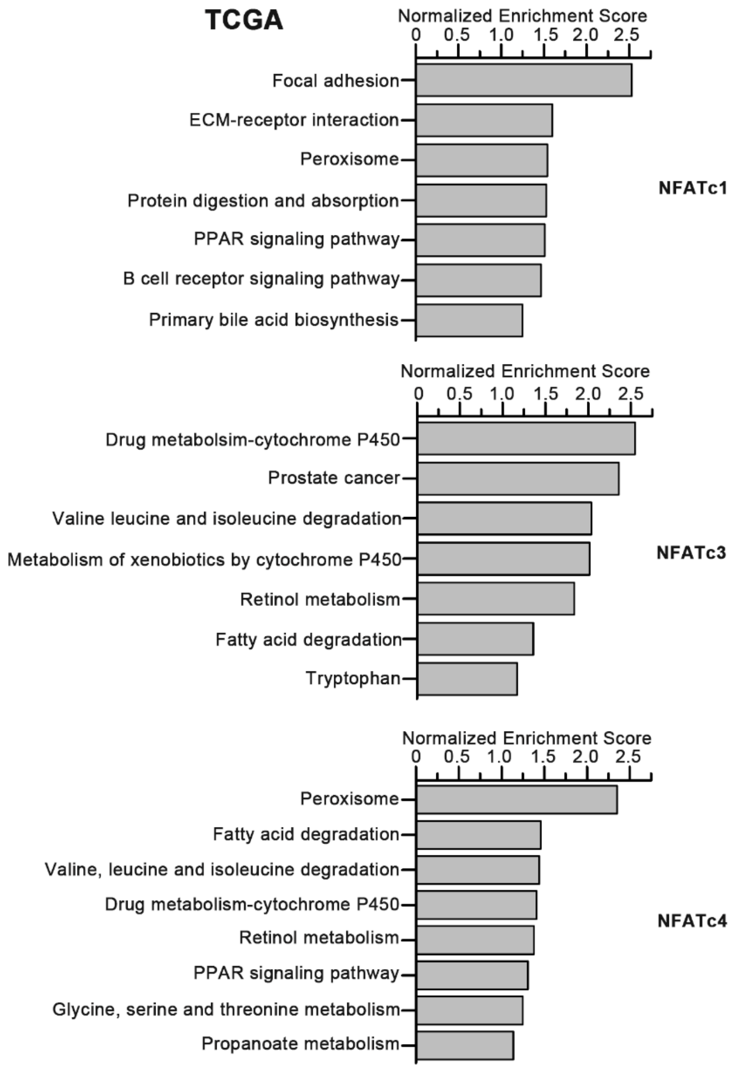

Figure S7. GSEA analysis of biological pathways associated with NFATc1, NFATc3 and NFATc4 in HCC. GSEA was performed to evaluate the biological pathways significantly associated with NFATc1, NFATc3 and NFATc4 in HCC using available micor-array data obtained from TCGA (n = 365)**.**

Figure S8

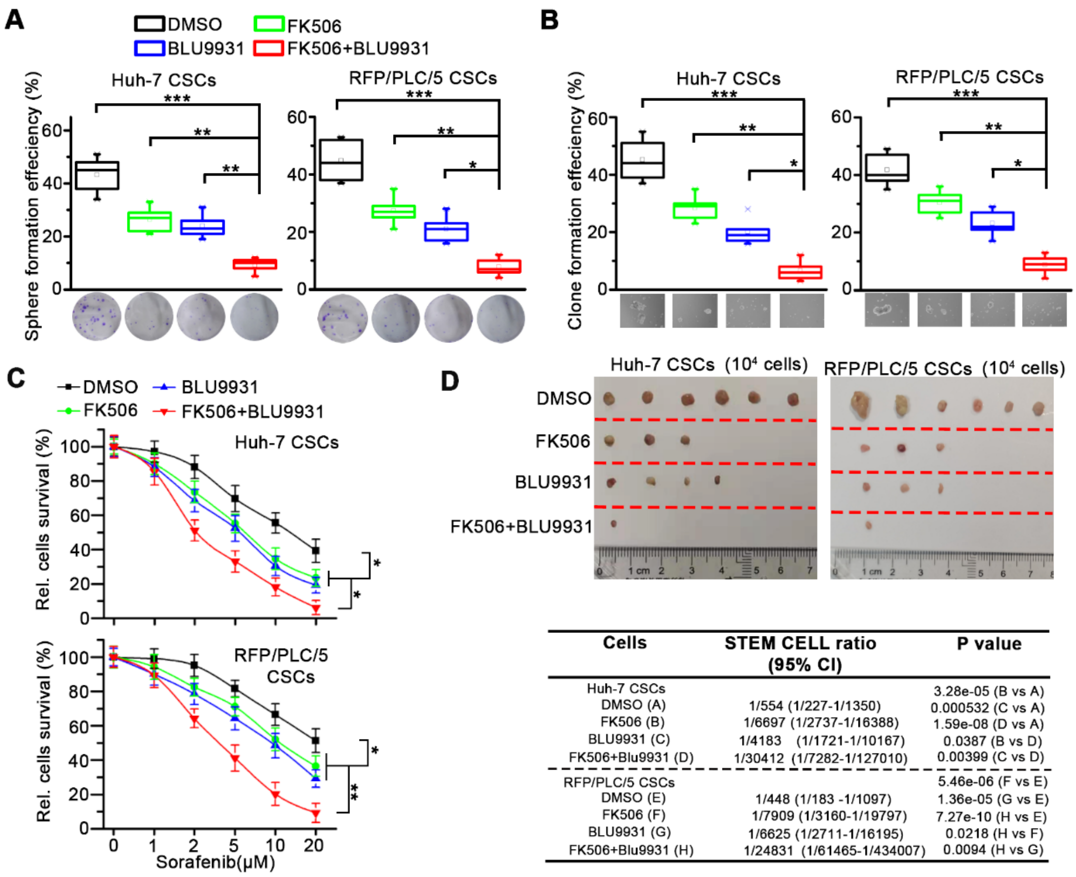

Cells	STEM CELL ratio (95% CI)	P value
Huh-7 CSCs		3.28e-05 (B vs A)
DMSO (A)	1/554 (1/227-1/1350)	0.000532 (C vs A)
FK506 (B)	1/6697 (1/2737-1/16388)	1.59e-08 (D vs A)
BLU9931 (C)	1/4183 (1/1721-1/10167)	0.0387 (B vs D)
FK506+Blu9931 (D)	1/30412 (1/7282-1/127010)	0.00399 (C vs D)
RFP/PLC/5 CSCs		5.46e-06 (F vs E)
DMSO (E)	1/448 (1/183 -1/1097)	1.36e-05 (G vs E)
FK506 (F)	1/7909 (1/3160-1/19797)	7.27e-10 (H vs E)
BLU9931 (G)	1/6625 (1/2711-1/16195)	0.0218 (H vs F)
FK506+Blu9931 (H)	1/24831 (1/61465-1/434007)	0.0094 (H vs G)

Figure S8. Combined administration of FK506 and BLU9931 attenuates self-renewal characteristics of LCSCs. The effects of BLU9931 (100 nM) alone, FK506 (50 nM) alone, or the combined administration of BLU9931 (100 nM) and FK506 (50 nM) on the (**A**) sphere formation assay, (**B**) clonogenicity assay, (**C**) sorafenib resistance assay and, and (**D**) tumorigenic potential assays *in vivo* in CSCs of Huh-7 and RFP/PLC/5. Data are expressed as means ± SEM (n = 3). *p < 0.05, **p < 0.01, ***p < 0.001.

Supplementary tables

Table. S1 Primers for RT-qPCR.

Name	Sequence of forward primer	Sequence of reverse primer
FGF19	GGAGATCCGCCCAGATGGCTAC	GGCCTCCAGTCCGGTGACAAGC
FGFR4	GGCCTCCAGTCCGGTGACAAGC	CCACAGCGTTCTCTACCAGG
NANOG	CAGAAGGCCTCAGCACCTAC	ATTGTTCCAGGTCTGGTTGC
OCT4	CAGTGCCCGAAACCCACAC	GGAGACCCAGCAGCCTCAAA
SOX2	GCACATGAACGGCTGGAGCAACG	TGCTGCGAGTAGGACATGCTGTAGG
ALB	CACAAAGATGACAACCCAAACCTCC	GGAGTTCCGGGGCATAAAAGTAAG
G6P	GTCTGTCACGAATCTACCTTG	CTACACCCAGTCCCTTGAG
CD133	AGTCGGAAACTGGCAGATAGC	GGTAGTGTTGTACTGGGCCAAT
MYC	TCAAGAGGCGAACACACAAC	GGCCTTTTCATTGTTTTCCA
ALDH1	GCACGCCAGACTTACCTGTC	CCTCCTCAGTTGCAGGATTAAAG
STIM1	TTGTCCATGCAGTCCCCTAG	GGTAGTGGTGATGGTGGTGA
STIM2	AGACAACAATGTCAAAGGAACGA	ACTCCGGTCACTGATTTTCAAC
ORAI1	GGACGCTGACCACGACTAC	GGGACTCCTTGACCGAGTT
NFATc1	GAGCCGAATGCACATAAGGTC	CCAGAGAGACTAGCAAGGGG
NFATc2	CACCGCATCACAGGGAAGAC	GCACAGTCAATGACGGCTC
NFATc3	CTTCTCCGATGCCTCTGACG	CGGGGCTTGGACCATACAG
NFATc4	GCTCGACTTCAAACTCGTCTT	GATGCACAATCATCTGGCTCA
GAPDH	AGCCACATCGCTCAGACAC	GCCCAATACGACCAAATCC

Table. S2 Antibody for WB and Immunohistochemistry.

Name	Application	Supplier	Cat no.	Clone no.
FGF19	WB, IHC	Cell Signaling Technology	Ca# 83348	D1N3R
FGFR4	WB	Cell Signaling Technology	Ca# 8562	D3B12
NFATc2	WB, IHC	Cell Signaling Technology	Ca# 5861	D43B1
Nanog	WB, IHC	Cell Signaling Technology	Ca# 4903	D73G4
Oct-4	WB, IHC	Cell Signaling Technology	Ca# 2750	N/A
Sox2	WB	Cell Signaling Technology	Ca# 3579	D6D9
STIM1	WB	Cell Signaling Technology	Ca# 5668	D88E10
STIM2	WB	Cell Signaling Technology	Ca# 4917	N/A
Orai1	WB	Abcam	Ca# ab244352	N/A
PLCγ	WB	Cell Signaling Technology	Ca# 5690	D9H10
p-PLCγ	WB	Cell Signaling Technology	Ca# 14008	D6M9S
p-ERK1/2	WB	Cell Signaling Technology	Ca# 9106	E10
ERK1/2	WB	Cell Signaling Technology	Ca# 9102	N/A
GAPDH	WB	Cell Signaling Technology	Ca# 5174	D16H11
α-Tubulin	WB	Beyotime	Ca# AF5012	N/A
Lamin B	WB	Beyotime	Ca# AF1408	N/A
Rabbit IgG	ChIP, IHC	Cell Signaling Technology	Ca# 3900	D15F1
NFATc1	WB	Cell Signaling Technology	Ca# 8032	D43B1
NFATc3	WB	Cell Signaling Technology	Ca# 4998	N/A
NFATc4	WB	Cell Signaling Technology	Ca# 2188	31G6
p-NFATc2 (Ser53)	WB	Affinity	Ca# AF3882	N/A
ALB	WB, IHC	Proteintech	Ca# 66051	4A1C11
G6P	WB	Abcam	Ca# ab207327	EPR20195
CaMKII-α	WB	Cell Signaling Technology	Ca# 3357	N/A
p-CaMKII (Thr286)	WB	Cell Signaling Technology	Cat#12716,	D21E4

Table. S3 Primers for ChIP-PCR.

Name	Sequence of forward primer	Sequence of reverse primer	Product
NFAT-RE1	GCTTCTCGGCTGGAGGGTGGT	GCAGGGACTCGGGGACTCAAAA	-911 ~ -792
NFAT-RE2	CACCACCCTCCAGCCGAGAAGC	GGCGGGGAAACAATGGAAGCC	-814 ~ -583
NFAT-RE3	GCAAGAAGATGAAGCTGAAAGAACCT	AAAAGCCCACTCGCACTCCC	-515 ~ -391
NFAT-RE4	CCCGAGGTTCTTGGCTGGGAGA	GATGTGCGGGGCTGCGAAAG	-439 ~ -175
